# Influence of very high temperatures on properties of medium grained lightweight aggregate mortars containing perlite and various cement types

**DOI:** 10.1038/s41598-025-29595-x

**Published:** 2026-01-12

**Authors:** Jan Pizoń, Miroslav Mynarz, Lucie Mynarzová

**Affiliations:** 1 Faculty of Civil Engineering, Silesian University of Technology, Akademicka 5, Gliwice, Poland; 2 Faculty of Safety Engineering, VŠB-Technical University of Ostrava, Lumírova 630/13, Ostrava-jih, Czech Republic; 3 Faculty of Civil Engineering, VŠB-Technical University of Ostrava, Ludvíka Podéště 1875/17, Ostrava-Poruba, Czech Republic

**Keywords:** Ordinary portland cement, Calcium sulphoaluminate cement, Calcium aluminate cement, Perlite, Lightweight cement mortars, High temperatures, Engineering, Materials science

## Abstract

This article examines the effects of very high temperatures (300–1000 °C) on the physical, mechanical, and thermal behaviour of medium-grained lightweight mortars incorporating expanded perlite (EP) and three cement types: Ordinary Portland (OPC), Calcium Sulphoaluminate (CSAC), and Calcium Aluminate (CAC). Mortars with 0–100% EP replacement were tested for compressive strength, density, absorbability, thermal conductivity, and microstructure. Increasing EP content reduced density and strength but enhanced thermal insulation and dimensional stability. Above 650 °C, OPC and CSAC underwent dehydration, ettringite loss, and microcracking, while CAC developed stable phases that preserved cohesion and colour. Strong correlations were found between thermal conductivity and compressive strength, allowing predictive modelling across temperatures. Polynomial correlations best described the strength–temperature relationship for OPC and CAC, while logarithmic models were optimal for CSAC. Overall, CAC-EP mortars demonstrated superior thermal stability, mechanical retention, and durability, highlighting their suitability for refractory and energy-efficient construction applications.

## Introduction

Lightweight aggregates offer significant benefits in concrete applications from both the economic and the environmental perspective. Their unique properties contribute to reduced material cost, enhanced structural performance, and improved sustainability in construction practices. Lightweight aggregates reduce the overall weight of concrete, which in turn decreases the dead load on structural elements. This reduction can lead to savings in foundation cost and material consumption. Additionally, the lower density of lightweight concrete allows for easier handling and transportation, further reducing logistic costs associated with construction^1,2^. Furthermore, lightweight concrete exhibits superior thermal insulation properties due to the air trapped within its porous structure, which can lead to reduced energy consumption for building interior heating and cooling^1,3^. This energy efficiency contributes to lower greenhouse gas emissions over the lifecycle of the buildings, aligning with global sustainability goals^4^.

Another advantage of structural elements made of lightweight concrete may be increased fire resistance or a lower tendency to explosive spalling. Temperature curves are usually used to assess the behaviour of structural elements in the event of fire, i.e. fire resistance, according to EN 1993-1-2. The most commonly used is the standard time-temperature fire curve (ISO 834), according to which most experimental tests of materials for fire protection are carried out. This curve corresponds to a fully developed fire in a closed space and its course starts at 20 °C and reaches approximately 1000 °C after 90 min of thermal exposure. Values ​​above 1000 °C are not very applicable in ordinary fires.

The present work expands previous research on fine-graded perlite-based lightweight cement mortars exposed to high temperatures^5^. In this paper, medium-grained expanded perlite (EP) as a lightweight aggregate for mortar is the research subject. In its natural state, perlite is a volcanic glass rock dominated by silica SiO_2_. Expanded perlite is prepared by heat treatment of raw perlite at a temperature in the range of 760–1100 °C^6^, which causes the water trapped within the glassy structure to vaporize, resulting in a significant expansion – up to 20 times its original volume. The expansion creates a unique structure characterized by numerous tiny, sealed air cells, contributing to its low density and high porosity, typically exceeding 90%^7^. The final product is lightweight, with a loose bulk density often less than 150 kg/m^3^, and displays low thermal conductivity (0.04–0.06 W/m K)^8^. The resulting material is obtained without additional chemical treatment. EP is used not only due to its lightweight, but also thanks to its thermal insulation properties, sound insulation, or good fire resistance^9^. Perlite can increase thermal resistance of various building materials^10^. Although, in most cases, EP may reduce mechanical strength of the material^11^, its advantages at elevated temperatures make it a valuable additive in high-temperature applications. According to^12^, properties of mortar with EP are significantly influenced by its amount, but also by the presence of other admixtures.

Perlite can also be added to fiber cement composites. However, if the composites contain a large amount of EP, then according to^13^ there is a significant reduction in mechanical properties of these materials at high temperatures. On the other hand, perlite aggregates provide better heat resistance compared to silica sand. This is due to the higher specific heat and lower thermal conductivity of perlite. Decrease of compressive strength of high-early-strength cement with perlite powder is also confirmed by^14^. This paper presents the development of a constitutive model for cement with perlite constrained by a carbon fiber-reinforced polymer. However, the thermal softening parameter implies the material behavior at elevated temperatures.

There are also other applications of perlite in similar building materials. After its thermal treatment it is ground to powder form called calcined perlite. The effect of calcined perlite on alkali-activated slag mortars was also investigated. As reported in^15^, the initial flexural and compressive strengths decrease with increasing perlite content, while the residual strengths at elevated temperatures improve significantly. This indicates that calcined perlite can increase heat resistance of these mortars.

Lightweight concrete and mortar can also contain aggregates other than expanded perlite, either natural or artificially produced (usually as by-products, e.g. fly ash or plastic waste aggregates). Expanded clay aggregate is a lightweight aggregate produced by heating clay to a temperature of approximately 1200 °C in a rotary kiln. During heating, the gases locked in the clay expand, creating thousands of tiny bubbles and giving the material its porous texture. Lightweight concrete with expanded clay aggregate achieves the highest strength and the lowest water absorption, as experimentally demonstrated in^16^. According to a review by^17^, ceramic aggregate in concrete improves workability, fire resistance, sound and thermal insulation. On the other hand, the same review mentions a reduction in density, strength and frost resistance of concrete with expanded clay aggregate. Pumice, extrusive volcanic rock formed by the outpouring of lava with a very high water and gas content from a volcano, is another aggregate that can be incorporated in lightweight concrete. Vermiculite is a flake-shaped mineral that increases its volume (expands) several fold using exfoliation technology. Exfoliated vermiculite and/or pumice were used to replace coarse or fine aggregate in^18^, where authors tested compressive strengths of samples with various amounts of these aggregates. Based on literature data, the authors of^18^ propose a model describing the relationship between the lightweight aggregate bulk density, the percentage replacement of natural aggregate and the resulting mechanical properties of the produced concrete. They note, for example, that cracks usually form in the grains of lightweight aggregate, not in the cement matrix.

The mechanical properties of mortars vary considerably depending on the type of cement used. This study investigates the behavior of mortars made from ordinary Portland cement (OPC), calcium sulphoaluminate cement (CSAC) and calcium aluminate cement (CAC) and provides insights into the optimization of cementitious materials coupled with EP with a particular attention to their behavior at elevated temperatures.

Ordinary Portland cement (OPC) is widely used in the construction industry due to its outstanding binding properties and availability. It is primarily made from calcium silicates and aluminates; its hydration produces calcium silicate hydrate and calcium hydroxide, which provide strength and durability to concrete and mortar. Its predictable performance, workability and cost-effectiveness make it a popular construction raw material. Nevertheless, OPC composites show a high thermal conductivity, which limits their suitability for thermal insulation. Although elevated temperatures accelerate the hydration process, initially leading to a denser microstructure, prolonged exposure usually leads to coarser porosity and reduced strength^19–22^. Mechanical strength is reduced due to thermal decomposition of its hydrated phases when exposed to high temperatures, which causes a problem in environments with fire risk or extreme temperatures. Together with mechanical strength, OPC concrete displays a weight loss due to moisture evaporation and dehydration of the products of hydration^23^. OPC concrete is also susceptible to thermal shock due to rapid temperature changes, which can cause cracking. The differences in thermal expansion between the concrete paste and the aggregates can lead to internal stresses, resulting in surface cracking and spalling^24,25^. The type of aggregates used in OPC concrete can significantly influence its behavior at elevated temperatures. Aggregates with low thermal expansion coefficients tend to perform better under high temperatures, while those with high expansion coefficients may exacerbate cracking and strength loss^25^. This is especially observed in connection with lightweight aggregate use and for this reason OPC composite was coupled with EP in this research.

Calcium sulphoaluminate cement (CSAC) is characterized by rapid hardening and lower environmental impact compared to OPC. However, its performance at elevated temperatures is less favorable, as studies indicate poor strength at high temperatures, making it unsuitable for applications requiring high thermal resistance. The hydration process of CSAC is heavily influenced by curing temperatures^26,27^, with higher temperatures accelerating hydration but causing variability in activation energy and hydration products. Despite its environmental advantages, CSAC’s mechanical properties degrade at elevated temperatures^28,29^, limiting its use in high-temperature environments. Its behavior at high temperatures, particularly regarding thermal decomposition and hydration product stability, is still poorly understood. This study aims, among other things, to address this gap by assessing the mechanical properties of CSAC-based mortars with different EP content under varying temperatures.

Calcium aluminate cement (CAC) is a specialized cement known for its high chemical resistance and rapid strength development. This is due to its unique temperature-dependent hydration mechanism. Unlike OPC and CSAC, CAC has very good refractory properties^30^, making it ideal for environments exposed to high temperatures and harsh chemicals^31,32^. CAC displays excellent properties at temperatures up to 1000 °C^5,31,33^. Material properties of calcium aluminate cement concrete at various temperatures are also studied by other authors^34^. Research indicates that while some hydration products may undergo phase transformations at high temperatures, CAC generally retains a more stable microstructure compared to OPC^35^. The addition of silica fume and lightweight aggregates such as pumice can further increase its heat resistance and strength^36^. Despite its advantages, CAC is used less commonly than OPC due to its higher cost and complex hydration chemistry. In addition, CAC can lose strength over time due to the chemical transformation of its hydration products under certain heat and moisture conditions. This problem can be mitigated by changing the hydration phase, for example by adding silica fume. This research investigates the performance of CAC-based mortars when combined with expanded perlite and subjected to high temperatures, aiming to determine the optimal conditions for their use in thermally challenging environments.

**Fig. 1:**
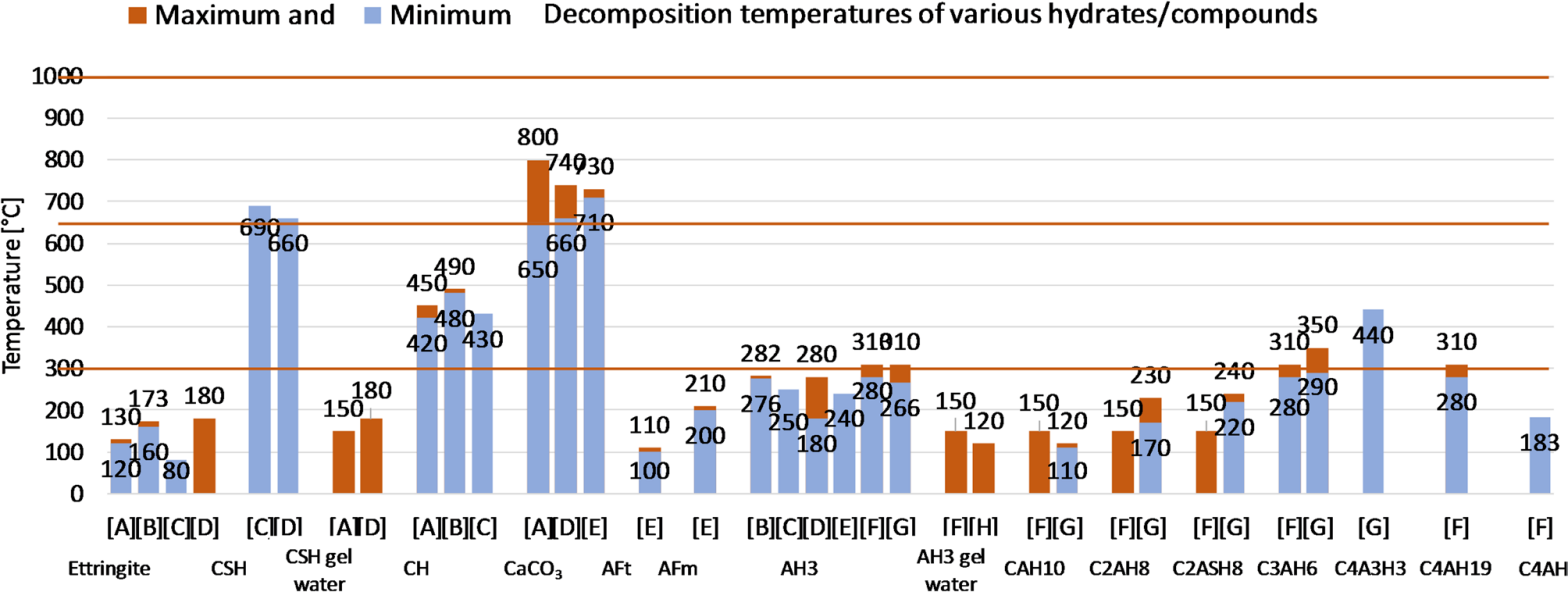
Decomposition temperatures of products of hydration: OPC, CSAS and CAC. Temperatures 300, 650, 1000 °C used in research are indicated with orange lines. (**A**)^40^; (**B**)^41^; (**C**)^42^; (**D**)^43^; (**E**)^44^; (**F**)^45^; (**G**)^46^; (**H**)^47^.

Thermal decomposition of hydrated cements is a complex process that involves multiple stages and mechanisms, influenced by cement composition and heating conditions. This is a critical factor in determining durability and properties of cementitious materials in high temperature environments^37,38^. When hydrated cements are exposed to elevated temperatures, water can be displaced from the products of hydration, leading to their microstructure breakdown. Understanding these processes^39^ is essential for the design of mortars that can withstand high temperature conditions without a significant loss of mechanical properties. Figure 1 shows decomposition temperatures of hydration products, OPC, CSAS and CAC. The temperatures to which the subject cement mortars were heated, and at which their properties were tested, were selected on the basis of the thermal decomposition of cement hydrates. These ranges are described in the “methodology” section. The temperatures at which the hydrates of individual cements decompose are given as ranges taken from the literature review. Some are relatively broad due to the fact that many factors influence the decomposition temperature of a given hydrate. These include the phase composition of the cement, the conditions during hydration (temperature, pressure), the composition of the composite (in particular the water-cement ratio and the content of supplementary cementitious materials and admixtures), the procedures for heating and testing samples, and the rate of heating and cooling.

## Novelty and perspectives for future research

Previous research focused on fine grained perlite (0–1 mm). In this research, a coarser type (0–2 mm) was used. The study was supplemented with several different tests and observations in addition to tests previously conducted on finer expanded perlite. This research also covers:

- SEM observations coupled with EDS analysis,
- volumetric and linear dimension changes,
- thermal properties and its relation to strength.

Additionally, an analysis of the best correlation model for compressive strength—temperature was performed.

In the course of the current study, some ideas for future research emerged, with two of them specifically worth a deeper analysis:

Porosity and pore size distribution study in mortars containing different cements and EP contents. This analysis should involve Mercury Intrusion Porosimetry (MIP) and image analysis.

Preparation of more accurate correlation models (by increasing the number of measurement points) between compressive strength and thermal conductivity for mortars after heating to 650 °C and 1000 °C.

### Materials

The study was conducted using cement mortars consisting of three different cements as binders. These were: Ordinary Portland cement (OPC), Calcium Sulphoaluminate Cement (CSAC) and Calcium Aluminate Cement (CAC). The detailed phase composition of the cements is shown in Table 1. Here, significant differences in composition can be seen. OPC is mainly composed of alite, belite, celite and brownmillerite, CSAC of ye’elemite, anhydrite and bredigite, and CAS of krotite and brownmillerite. These differences affect the composition and properties of the resulting composites, in particular the different decomposition temperatures of the various hydrates described in the introduction. Each cement was mixed with 10% of silica fume.

**Table 1:** Phase composition of cements.

Cement		OPC	CSAC	CAC
Phase				
C_3_S	Alite	71.1		
C_2_S	Belite	5.6	4.8	
C_3_A	Celite	3.9		5.2
C_4_AF	Brownmillerite	12.7	2.6	19.1
C_4_A_3_Ŝ	Ye’elemite		43.5	
CŜ	Anhydrite		16.4	
C_7_MS_4_	Bredigite		11.9	
MgO	Periclase	0.15	3.7	
C_3_MS_2_	Merwinite		5.9	
CaCO_3_	Calcite	4.3		
CA	Krotite			59.9
C_12_A_7_	Mayenite			1.7
C_2_AS	Gelhenite		2.7	8

**Fig. 2:**
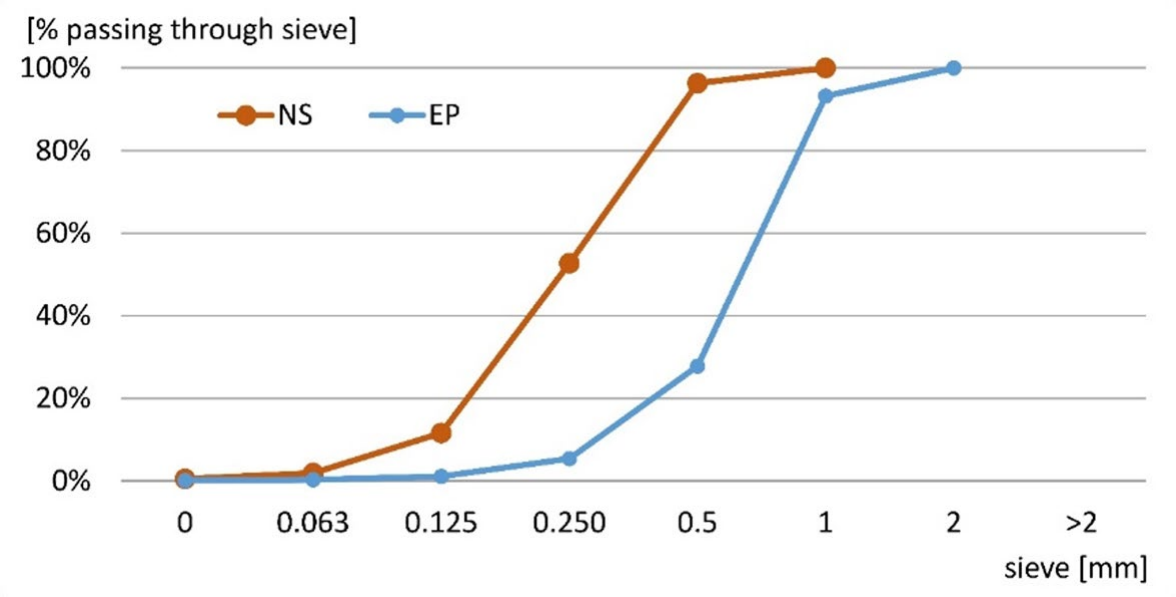
Grading curve of natural sand (NS) and expanded perlite (EP).

The aggregates used to prepare the mortars included sand and expanded perlite (EP). The chemical composition of EP is shown in Table 2. The bulk density is 1651.2 g/dm^3^ and 102.3 g/dm^3^ for sand and EP, respectively. The real density is 2652 g/dm^3^ and 2320 g/dm^3^ for sand and EP, respectively. The grading curves of the two aggregates are shown in Fig. 2.

**Table 2:** Chemical composition of expanded perlite [%].

SiO_2_	Al_2_O_3_	K_2_O	Na_2_O	MgO	CaO	Fe_2_O_3_
74.5	13.9	4.2	4.4	0.6	1.0	1.1

**Fig. 3:**
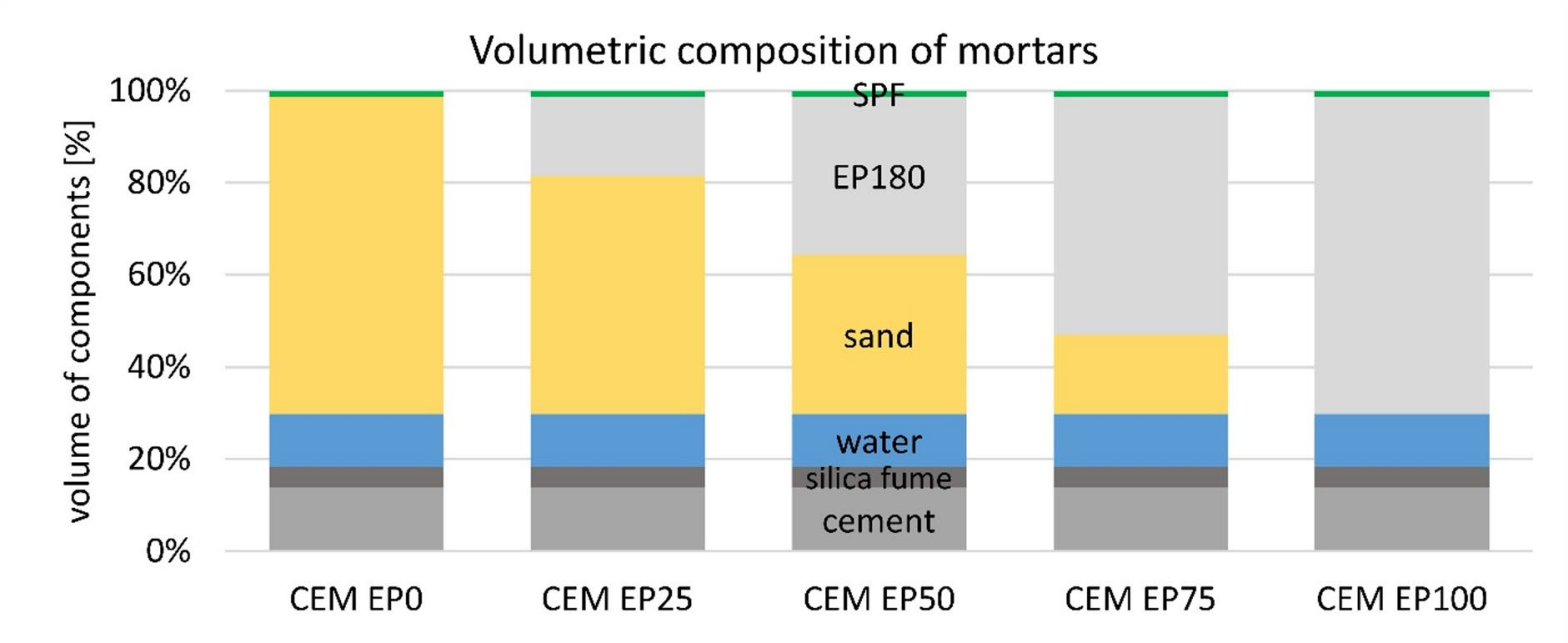
Volumetric composition of mortars. CEM denotes OPC or CAC or CSAC.

**Fig. 4:**
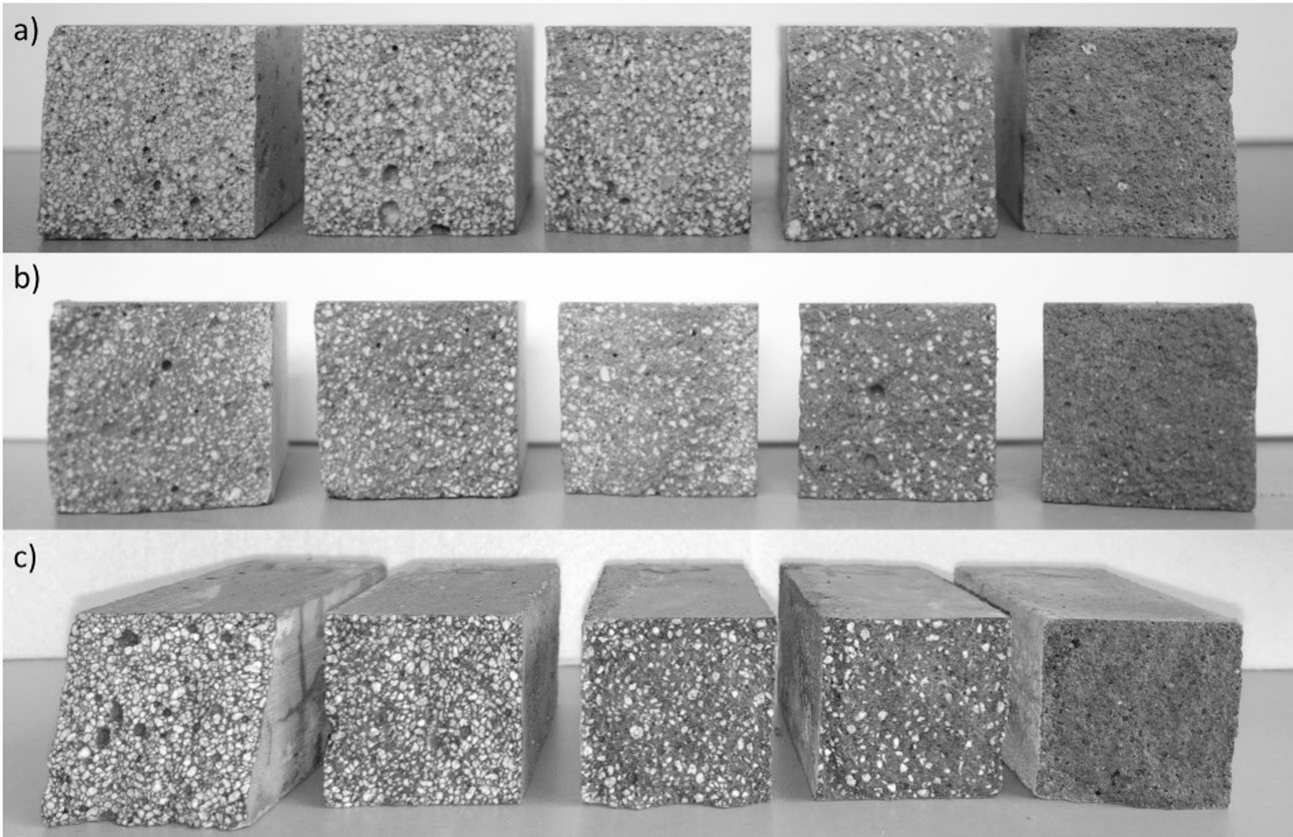
Cross-sections of mortar samples containing (**a**) OPC, (**b**) CSAC, (**c**) CAC, and EP content decreasing from left (100%) to right (0%).

The mixtures for the present research were designed as a combination of a binder in a constant proportion, tap water with a set water/cement ratio of 0.5, a mix of aggregates and a superplasticizer. Specific mass composition of mixtures is presented in Table 3. The aggregates were mixed and the EP substituted sand in 0%, 25%, 50%, 75% and 100% by volume. The volumetric composition is presented in Fig. 3. Superplasticizer (SP) was added in the amount necessary to obtain a constant consistency of 14 ± 1 cm. In order to illustrate the differences in EP content in relation to the volume of mortar, Fig. 4 was prepared, showing hardened mortar samples made from different cements with different EP contents.

**Table 3:** Mortars composition by mass.

Symbol	Cement type, mass		Silica fume	Water	Expanded perlite (EP)	Sand	Superplasticizer (SP)	
		(g)	(g)	(g)	(g)	(g)	(g)	(% of cement mass)
OPC EP0	CEM I 42,5 R	1620	180	900 (w/b ratio 0.5)	0	8640	15	0.83
OPC EP25					553	6480	18	1.00
OPC EP50					414	4320	23	1.28
OPC EP75					276	2160	28	1.56
OPC EP100					138	0	33	1.83
CSAC EP0	Calcium sulphoaluminate cement				0	8640	7	0.39
CSAC EP25					553	6480	10	0.56
CSAC EP50					414	4320	14	0.78
CSAC EP75					276	2160	22	1.22
CSAC EP100					138	0	30	1.67
CAC EP0	Calcium aluminate cement				0	8640	16	0.89
CAC EP25					553	6480	18	1.00
CAC EP50					414	4320	23	1.28
CAC EP75					276	2160	28	1.56
CAC EP100					138	0	33	1.83

## Methods

### Fresh mix preparation and properties

All mortars were prepared under uniform conditions using the same methodology. The mortars were combined with a specific quantity of SP to achieve a consistent diameter of 14 ± 1 cm during the flow table test. This uniformity in compaction was essential for the subsequent testing phases. Initial trial batches were created to determine the appropriate dosage of SP. Initially, cement and water were blended. After one minute, SP was worked into the mixture, which was then mixed for an additional minute. Subsequently, sand was added to the bowl and mixed with the cement paste for one minute. Finally, EP was introduced, and the mixing duration ranged from one to one and a half minutes to ensure a homogeneous mixture with the desired consistency. The addition of EP occurred at the end of the mixing process to prevent crushing of its particles and to minimize excessive water absorption from the mixture. The flow table tests were performed in accordance with the EN 1015-3 standard. The variation in consistency was measured by the dosage of SP used. The density of the fresh mixtures was determined following the guidelines set forth in the EN 1015-6 standard.

### Hardened mortar preparation

**Fig. 5:**
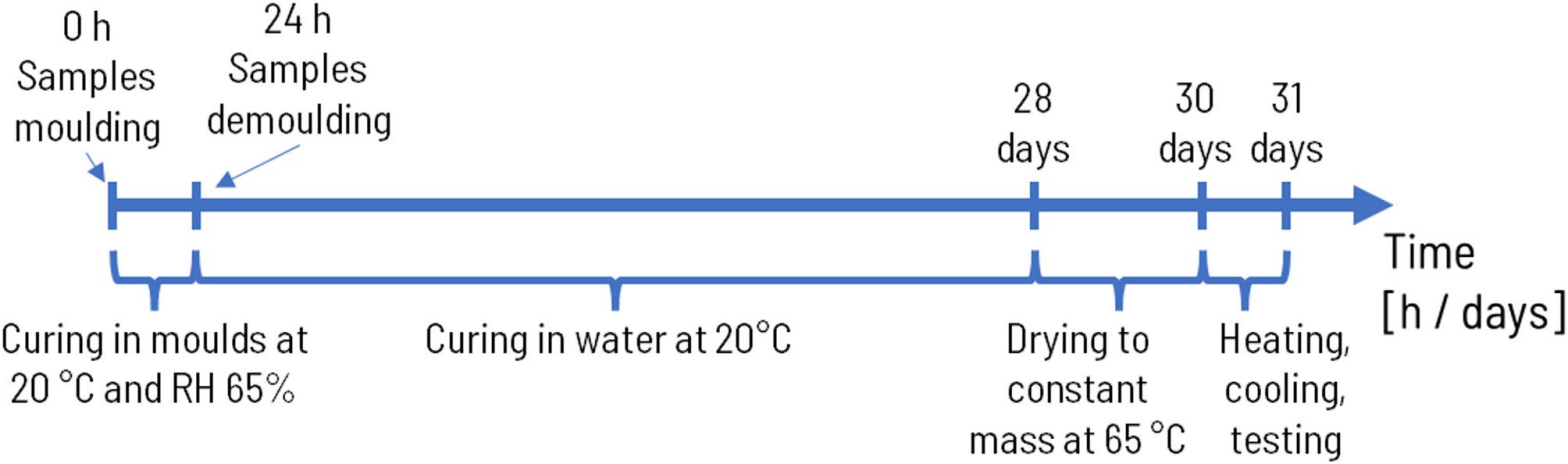
Preparation process of mortar samples in the form of timeline.

**Fig. 6:**
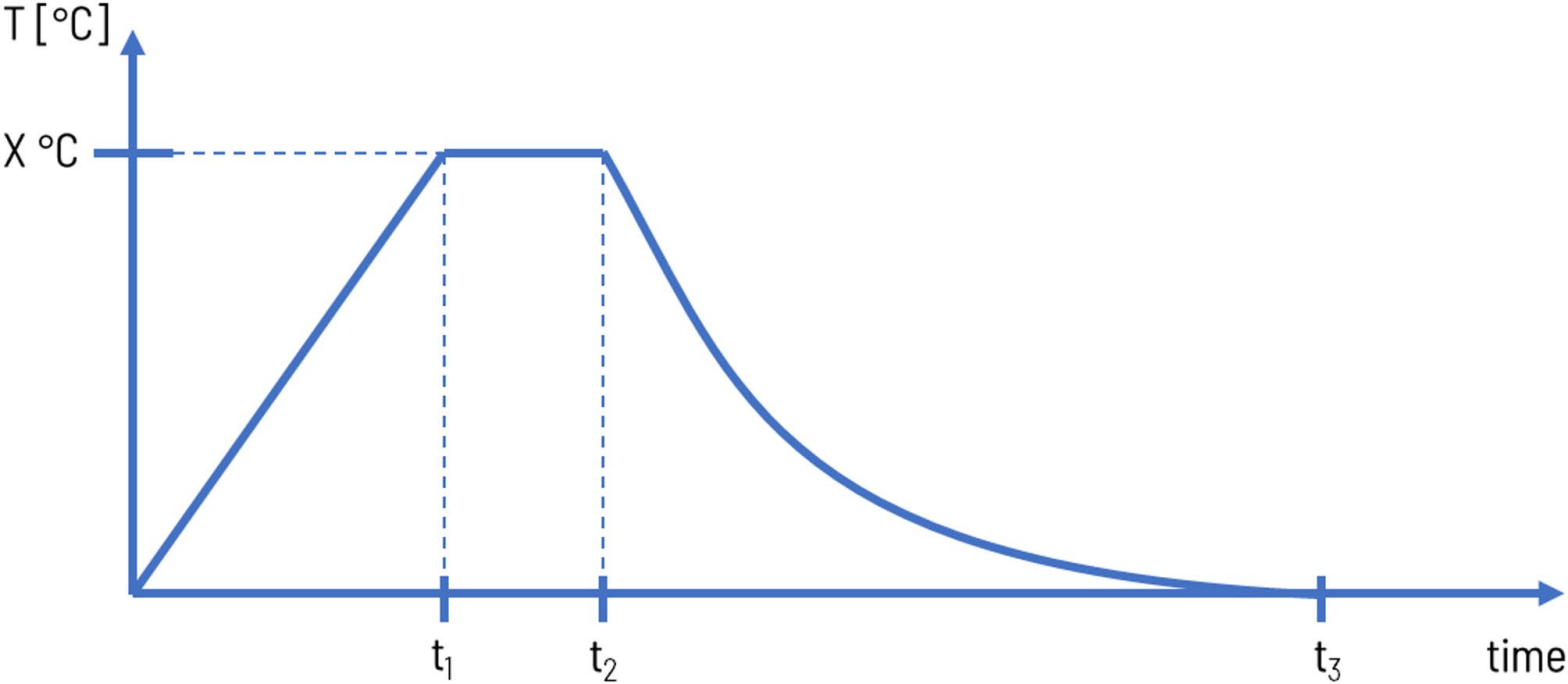
Heating procedure: t_1_ – time of heating of furnace to T = X °C @ rate 300 °C/h; t_2_ = t_1_ + 60 min @ stable T = X °C; t_3_ – time of cooling of furnace to room temperature (non-linear); X = 300, 650, 1000 °C.

Samples of hardened mortars intended for testing of compressive strength, density, absorbability, thermal properties and SEM observations were prepared, cured, and evaluated in accordance with the EN 196-1 standard. After 24 h of curing in moulds at 20 °C and at a relative humidity of 65%, the mortars were submerged in a water bath maintained at 20 °C for 27 days. Following this curing period, the samples were removed and dried at 65 °C until they reached a constant mass. The temperature was set as 65 °C to prevent gypsum dehydration. Once dried, the samples were placed in an electric furnace, with specimens separated by chamotte dividers to prevent them from fusing together. The samples were subjected to heating at temperatures of 300, 650, and 1000 °C, with a heating rate of 300 °C per hour. Isothermal conditions at each specified temperature were maintained for one hour. The cooling process was non-linear, with the samples cooling down together with the furnace. The details of the preparation and heating processes are summarized in Figs. 5 and 6.

The selection of temperatures is justified by Fig. 1, which can be found above in the introduction, and further clarification is provided below:

- 300 °C—This temperature is at or above that of the end of the decomposition of aluminum hydroxide (AH3) and the majority of other hydration products of calcium aluminate cement (CAC), as well as above the decomposition temperature of aluminum ferrite monosulfate AFm and ettringite.
- 650 °C—This temperature exceeds that of the decomposition of calcium hydroxide (CH) and is at or above this point for calcium silicate hydrate (CSH).
- 1000 °C—This temperature is above that of CaCO_3_ decomposition and in range of typical perlite softening (871–1093 °C), but below its melting point (~ 1260–1343 °C)^48^.

### Hardened mortar properties

Following the cooling process, the samples were measured, weighed, photographed, and subjected to compressive strength testing on six samples of each type. Confidence intervals are illustrated in the accompanying diagrams. Their compressive strength was evaluated in accordance with the EN 196-1 standard, while density measurements were according to the EN 1015-10 standard, and absorbability was assessed using the procedure described in the EN 13755 standard (this was only conducted for samples prior to heating).

Photographs of the samples were taken after they were heated to 650 and 1000 °C, but before the strength tests were performed, in order to document the thermal cracks that were visible on the surface. No observable changes were detected by the naked eye in samples heated to 300 °C.

A Scanning Electron Microscope (SEM) model JEOL JSM-7200 F (JEOL company, Tokyo, Japan) was used to perform microstructure observations. It was equipped with an Energy-dispersive X-ray spectroscopy (EDS) analyzer.

The thermal properties were evaluated using the ISOMET 2114 Portable Thermal Properties Analyzer (Applied Precision s.r.o., Bratislava, Slovakia). The samples utilized for these assessments were cubic in shape, measuring 10 × 10 × 10 cm. The determination of thermal properties with the ISOMET device employs the ‘hot plate’ technique, which involves monitoring changes in the surface temperature of the sample during two distinct phases. The first phase occurs while the sample is heated at a constant power level, and the second phase takes place during the cooling process. The recorded measurements included thermal conductivity λ [W/(m∙K)] and volumetric heat capacity cρ [J/(m^3^∙K)]. The compressive strength tests were conducted on 6 samples of each type, the rest using only one sample of each type.

## Results and discussion

### Properties of fresh mixes

**Fig. 7:**
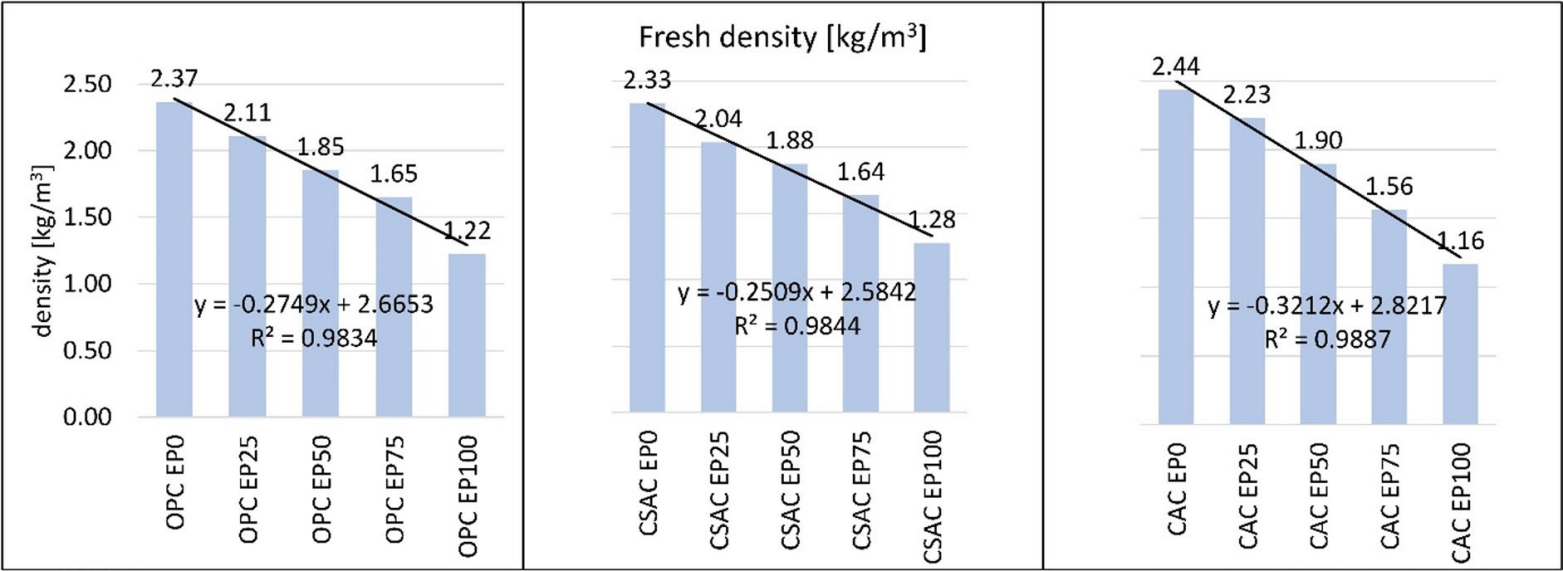
Density of fresh mortars.

Figure 7 presents a summary of the densities of mortars manufactured with different types of cement and various replacement ratios of EP. It is evident that the density decreases as the perlite content increases. Among mortars that solely utilized sand as the aggregate, the highest density was observed in those made with CAC, while the lowest density was found in mortars using CSAC. Conversely, when 100% EP was incorporated, the trend reversed, with CSAC exhibiting the highest and CAC the lowest density. Other density variations were noted for intermediate EP replacement levels. However, it is important to note that the observed differences in density are minimal and should not be regarded as statistically significant. The absorbability of the perlite itself is a reason why increasing amount of EP is not affecting the density in a more pronounced way. Water infiltrates the pores of the EP, which would otherwise be filled with air, thereby increasing the overall volume of the mixture. A potential method to mitigate this issue could involve pre-wetting the perlite using a portion of the water designated for mortar preparation (rather than adding extra water). This approach would not only provide an additional source of water for cement hydration but also help maintain constant consistency throughout the mixing process^10^. Studies have shown that using pre-soaked lightweight aggregates can also significantly reduce shrinkage and enhance the hydration of cement, leading to improved compressive strength and reduced permeability^49^.

**Fig. 8:**
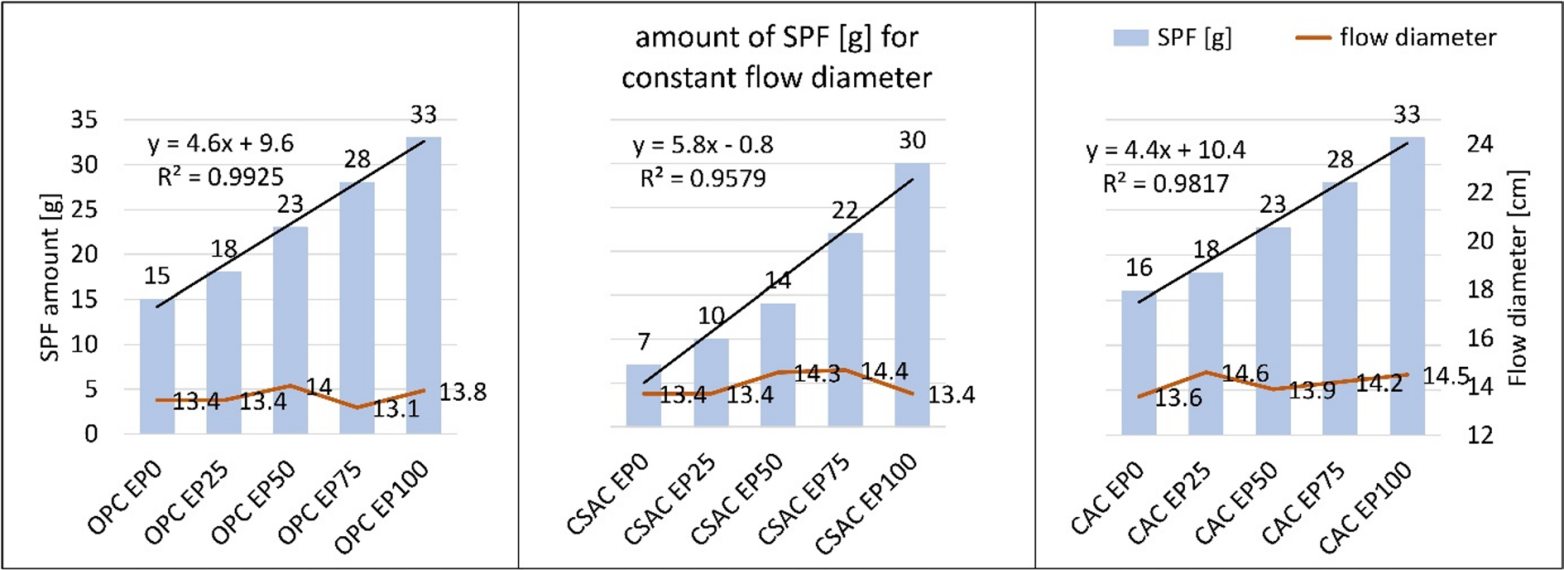
Superplasticizer amount for constant flow diameter.

Consistency of the fresh mixes was evaluated immediately after the mixing process. As illustrated by Fig. 8, the quantity of superplasticizer (SP) required to achieve a flow diameter of 14 ± 1 cm was nearly identical for both OPC and CAC mortars. Specifically, the amount of SP increased from 15 g for OPC and 16 g for CAC-based mortar when sand was used as the aggregate. Furthermore, the SP dosage increased with the amount of EP, reaching 36 g for OPC mortars and 38 g for CAC mortars when 100% perlite was incorporated. In contrast, the requirement for SP in CSAC mortars was lower, regardless of the EP content added. The increase in SP dosage required by OPC and CAC in comparison to CSAC ranged from 16% for mortars containing 100% perlite to as much as 55% for those containing sand only. This indicates that the primary factor influencing the consistency of the mix is the type of cement used when the perlite content is low, whereas the perlite itself becomes the dominant factor when its content is higher. The comparative behavior of OPC and CAC in relation to CSAC concerning consistency, viscosity, and rheological parameters is distinctly defined and influenced by the specific type, surface area, and composition of the cements involved^50^. This phenomenon is attributed to the rapid setting characteristics of CSAC, which complicates the standard testing procedure executions. Existing literature presents conflicting findings regarding the consistency of CSAC-based concrete, with some studies reporting superior performance and others indicating the opposite^51–53^. Additionally, CAC is known to lose workability swiftly over time^54^. It is noteworthy that there is a lack of reported results concerning CSAC with silica fume blends thereof, although it has been suggested that CAC-SF may exhibit improved consistency^55^.

The quantity of SP and the density of mortar in its plastic state exhibit a strong correlation with the EP content. For both analyses, linear trend lines were employed to describe the coefficient of determination. The coefficient of determination (R^2^) values for the relationship between perlite content and SP dosage ranged from 0.9579 to 0.9925, indicating a clear correlation. For the correlation of density and EP content, the R^2^ ranged between 0.98 and 0.99 for all cements. The relevant graphs can be found in Figs. 7 and 8. The equations presented in Figs. 7 and 8 should be considered as potential tools for predicting the density of mortars with intermediate levels of perlite. The increased water requirement of lightweight cement composites containing expanded perlite is closely associated with their porosity, which is a fundamental characteristic of lightweight aggregates. This relationship has been documented by multiple researchers across different lightweight materials^56,57^. Similar results for fresh mortar properties were obtained during previous research dealing with fine graded perlite^5^. This shows that the results are consistent regardless of fineness of EP within the tested range.

### Mechanical properties of mortars

**Fig. 9:**
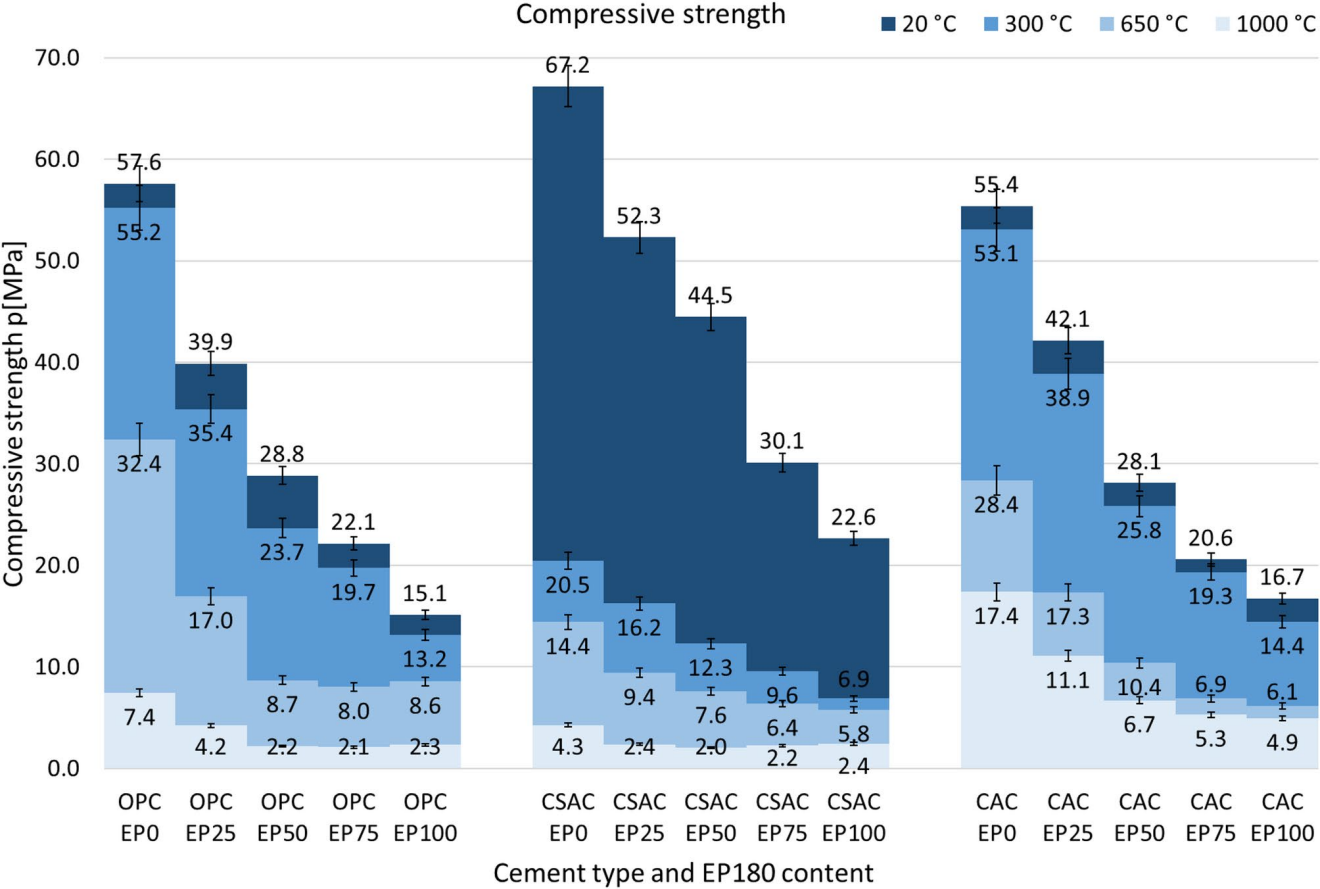
Compressive strengths of mortars subjected to various temperatures with values and confidence interval.

Compressive strength of the mortars was evaluated following the curing of all samples for 28 days in accordance with the EN 196-1 standard. Subsequently, selected samples were dried to a constant mass over a period of 48 h, after which compressive strength tests were performed. The remaining samples were exposed to elevated temperatures of 300, 650, and 1000 °C. Once the cooling process ended, compressive strength tests were conducted. Figure 9 presents the results of the mortar compressive strength tests. 

Among all mortars, those produced with CSAC, and not exposed to high temperatures, achieved the highest compressive strength of 67.2 MPa for the sample with no EP, and decreasing to 22.6 MPa for the sample with 100% EP content. In the comparison, the compressive strengths of the OPC and CAC mortars were similar, measuring 57.6 MPa and 55.4 MPa for samples without perlite, and 15.1 MPa and 16.7 MPa for those containing 100% perlite, respectively.

The overall behavior of mortars is consistent and predictable: an increase in perlite content leads to a decrease in compressive strength, and similarly, higher temperatures during heating also result in reduced strength. Notably, the compressive strengths of OPC- and CAC-based mortars are comparable up to 650 °C .

After exposure to 300 °C, the compressive strengths recorded are 55.2 MPa for OPC and 53.1 MPa for CAC for samples without perlite, while for those containing 100% perlite, the strengths are 13.2 MPa for OPC and 14.4 MPa for CAC. When samples without perlite are heated to 650 °C, their strengths decrease to 32.4 MPa for OPC and 28.4 MPa for CAC, whereas samples with 100% expanded perlite exhibit strengths of 8.6 MPa for OPC and 6.1 MPa for CAC. Both OPC and CAC mortars experience a significant loss of strength between the temperatures of 300 °C and 650 °C.

The disparity in strength becomes more pronounced after heating to 1000 °C. For OPC-based mortars, the residual strength is measured at 7.4 MPa for samples without perlite and 2.3 MPa for those with 100% perlite substitution. In contrast, CAC mortars demonstrate higher residual strengths of 17.4 MPa and 4.9 MPa for the respective sample types. This difference can be attributed to the superior heat resistance and refractory properties of the CAC composites, primarily due to the presence of calcium aluminate (CA) as the main phase in CAC^58^. It is also important to note that while CAC mortars undergo sintering at temperatures exceeding 1000 °C, which results in an increase in strength, this process already begins before reaching 1000 °C^46,59^. The importance of residual strength of concrete was recalled by several researchers, and it is believed to diminish not only by thermal decomposition of hydrates, but also due to extensive inner cracking, which is exacerbated at temperatures around 600–700 °C^60^, and may be at about 6% after exposure to 1200 °C^61^.

CSAC mortars exhibit distinct behavior. A significant reduction in compressive strength is observed at temperatures below 300 °C, which is attributed to the considerably higher ettringite content present in hydrated CSAC compared to OPC. In CSAC, ettringite plays a crucial role in strength shaping, whereas in OPC, it is the calcium silicate hydrate phase (CSH) that is primarily responsible for strength development. Notably, ettringite begins to thermally decompose at temperatures below 180 °C, while the CSH phase does not decompose until temperatures exceed 600 °C. The decomposition of the ettringite above 180 °C is not the only reason for the poor performance of CSAC mortars. The irreversible dehydration coupled with structural changes and formation of an amorphous metaettringite or a bassanite formation may occur during prolonged heating even in range of 50–100 °C^41–43^. Since the samples were dried for 48 h in 65 °C, it may attribute to its even poorer performance. Despite these differences, there is a similarity in the behavior of OPC and CSAC, particularly in their residual strengths after heating to 1000 °C. The residual strengths are well comparable, being 7.4 MPa for OPC and 4.3 MPa for CSAC for samples without perlite, and 2.3 MPa for OPC and 2.4 MPa for CSAC for samples containing 100% perlite. In contrast, the residual strength of CAC mortars is higher, recorded at 17.4 for sample without EP and 4.9 MPa for the one with 100% EP.

Considering the residual strength as a percentage of initial strength, it tends to be higher in samples with a lower perlite content. The smallest differences are observed in the case of CSAC. The similarity in behavior between OPC and CAC is evident across all perlite contents at temperatures up to 300 °C, where both cements exhibit similar strength drops. This similarity continues up to 650 °C. However, the behavior of CSAC diverges significantly from that of OPC and CAC. At 1000 °C, the highest residual strength being observed in CAC, with values in the range of 25–36%. In comparison, the residual strengths for OPC and CSAC are much lower, recorded at 7–14% and 7–8%, respectively.

Considering the behavior of individual mortars and their interrelationships between the various cements and EP contents, mortars containing finer EP (from previous studies^5^ show behaviors similar to the behavior of those used for the current study. The differences are apparent quantitatively, and mortars containing finer EP show higher strengths in groups of individual cements as well as when comparing the same amounts of finer and coarser EP.

Two different correlation analyses were performed. The first one setting the compressive strength against EP content at various temperatures separately, and the second one correlating compressive strengths and temperatures for different cement/EP content pairs.

**Fig. 10:**
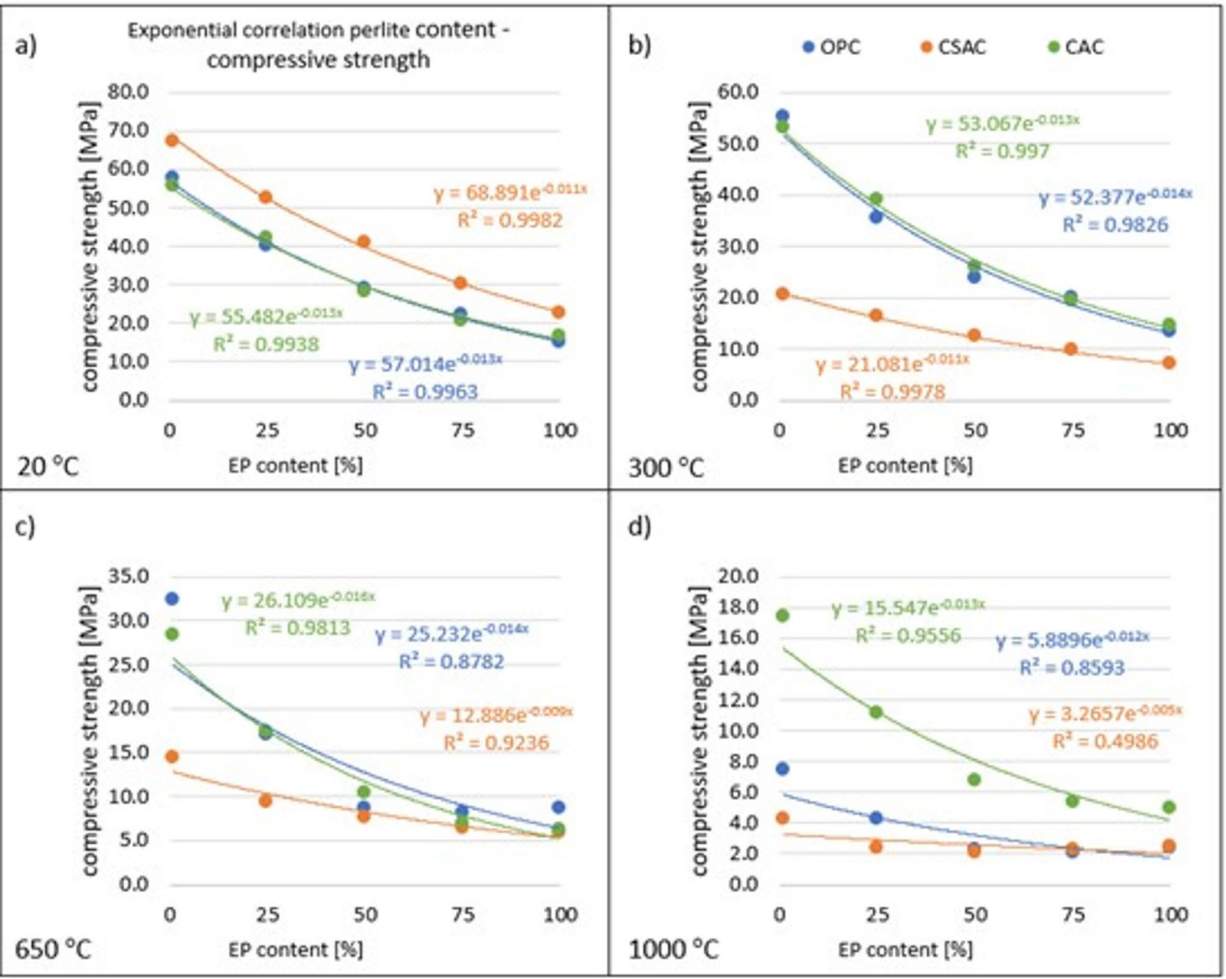
Correlations of EP content and compressive strength for COP, CSAC, CAC and for 20–1000 °C.

Figure 10 illustrates the relationships between compressive strengths and EP contents across all types of cement, demonstrating strong correlations. Exponential correlation was selected for this presentation, as it better represents the results compared to linear correlation. The non-linearity of this relation is partly supported by other research in which correlation is used where the compressive strength of lightweight aggregate concrete is expressed as a function of the aggregate density^62^, but not as a lightweight aggregate amount like in the present research.

The R^2^ coefficients for these correlations are about 0.99 for temperatures 20 °C and 300 °C. For higher temperatures the correlation is very strong in the case of CAC (R² values 0.95 for 650 °C and 0.98 for 1000 °C) and CSAC at 650 °C (R^2^ = 0.92), lower for OPC (R^2^ values 0.85 for 650 °C and 0.87 for 1000 °C), and the lowest for and CSAC at 1000 °C (R^2^ = 0.50). Using this model the behavior of CSAC in the last case is difficult to predict. In all other cases proposed models can be utilized for estimating the strength of mortars with intermediate perlite content, taking into account that those models may be a bit less effective for EP content near 0%, and 100%, which is visible in Fig. 10c) for OPC at 650 °C.

**Fig. 11:**
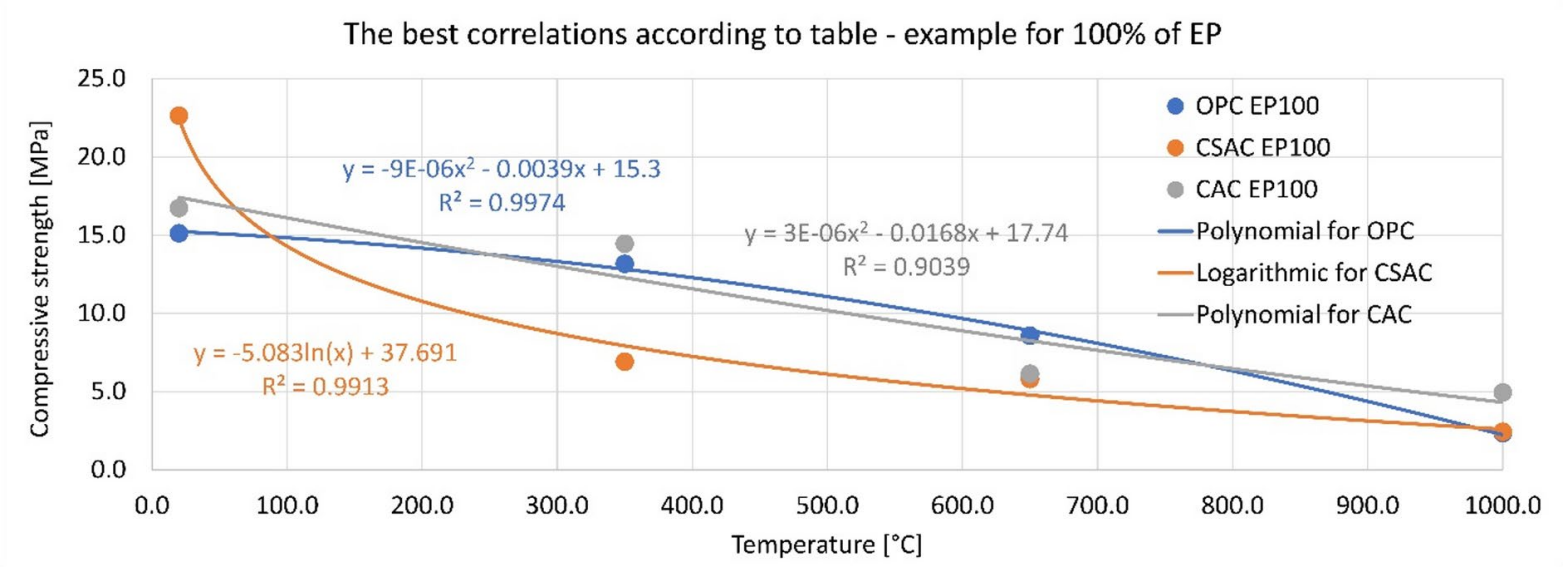
The best correlations for different cements. Example for 100% EP content. For the remaining results see Table 4.

The second analysis concerns the correlation of compressive strength with temperature, shown in Table 4. There are four different correlation models (linear, 2nd degree polynomial, exponential and logarithmic). The table shows the R^2^ values for each cement paired with EP content. The analysis demonstrates that for OPC and CAC, the polynomial correlation can be taken as the best representation of the strength-temperature relationship. For OPC, the R^2^ was between 0.947 and 0.997, and for CAC the R^2^ was between 0.857 and 0.924, indicating an excellent correlation. In both cases, linear correlation also yields a very good R^2^ of between 0.85 and 0.96. The exponential and logarithmic models yield worse results. The situation is different for CSAC. The logarithmic model gave an R^2^ value above 0.99 for each EP content. The second and third-best models are polynomial and exponential, always producing a value above 0.94. The linear correlation is weaker. This indicates a large divergence in the properties of the tested cements. Because of the large number of graphs to be presented, only an example of best correlations for 100% of EP is presented in Fig. 11.

**Table 4:** R^2^ for different models of compressive strength—temperature correlation.

Model	Linear	Polynomial (2nd degree)	Exponential	Logarithmic
OPC EP0	**0.9144**	**0.9833**	0.7016	0.5454
OPC EP25	**0.9516**	**0.9677**	0.7736	0.6470
OPC EP50	**0.9540**	**0.9542**	0.8053	0.7177
OPC EP75	**0.9392**	**0.9471**	0.7655	0.6515
OPC EP100	**0.9563**	**0.9974**	0.7911	0.6290
CSAC EP0	0.8126	**0.9631**	**0.9789**	**0.9964**
CSAC EP25	0.8262	**0.9758**	**0.9927**	**0.9991**
CSAC EP50	0.8157	**0.9709**	**0.9882**	**0.9986**
CSAC EP75	0.8157	**0.9692**	**0.9799**	**0.9981**
CSAC EP100	0.7842	**0.9482**	**0.9469**	**0.9913**
CAC EP0	**0.9101**	**0.9236**	0.8324	0.6025
CAC EP25	**0.9060**	**0.9074**	0.8392	0.6446
CAC EP50	**0.8952**	**0.8956**	0.8271	0.6444
CAC EP75	**0.8570**	**0.8571**	0.8020	0.6201
CAC EP100	**0.8987**	**0.9039**	0.8790	0.7107

### Physical properties of mortars

The first observation evident when the samples were removed from the oven was the change in their linear dimensions. The results of the measurements showing length change and percentage change can be found in Table 5. The table also demonstrates volumetric changes.

**Table 5:** Data showing linear and volumetric changes of samples after heating up to 650 °C and 1000 °C. The changes were not noticeable after heating up to 300 °C. Where there is no value in the table the decrease was unnoticeable.

	650 °C				1000 °C			
	ΔL	ΔH	ΔW	ΔV	ΔL	ΔH	ΔW	ΔV
	[cm] (%)			[cm^3^] (%)	[cm] (%)			[cm^3^] (%)
OPC EP100	0.3 (1.9)	0.1 (2.5)	0.1 (0.6)	17.2 (6.7)	0.7 (4.4)	0.2 (5.0)	0.2 (5.0)	35.1 (13.7)
OPC EP75	0.1 (0.6)			1.6 (0.6)	0.2 (1.3)	0.1 (2.5)		9.5 (3.7)
OPC EP50					0.1 (0.6)			1.6 (0.6)
OPC EP25								
OPC EP0								
CSAC EP100	0.3 (1.9)	0.1 (2.5)		11.1 (4.3)	0.4 (2.5)	0.2 (5.0)	0.1 (2.5)	24.8 (9.7)
CSAC EP75	0.1 (0.6)			1.6 (0.6)	0.2 (1.3)	0.1 (2.5)		9.5 (3.7)
CSAC EP50					0.1 (0.6)			1.6 (0.6)
CSAC EP25								
CSAC EP0								
CAC EP100	0.1 (0.6)	0.1 (2.5)		8.0 (3.1)	0.3 (1.9)	0.1 (2.5)	0.1 (2.5)	17.2 (6.7)
CAC EP75					0.1 (0.6)	0.1 (2.5)		8.0 (3.1)
CAC EP50								
CAC EP25								
CAC EP0								

The samples were 16 cm in length, and in this direction the changes were most pronounced. The shortening of the samples was only observed after heating to 650 °C and 1000 °C and increased with increasing EP content in the sample. In connection with heating to 650 °C, there is no clear difference between OPC and CSAC with 100% EP content. Both samples shrank by 0.3 cm (1.9%) in their largest dimension. Volume decreased by 17.2 cm^3^ and 11.1 cm^3^, or 6.7% and 4.3% for OPC and CSAC, respectively. For the EP content of 75%, shrinkage was minimal. At lower contents, it was not noticeable at all. After heating to 650 °C, the sample containing CAC and 100% EP shrank by 0.1 cm (0.6%) in length and its volume decreased by 8 cm^3^ (3.1%). For EP contents of 75% and less, no shrinkage was apparent. For heating to 1000 °C, the dimensional changes were more pronounced. In the case of heating to 1000 °C, the OPC samples containing 100% EP showed the highest shrinkage. The largest shortening was by 0.7 cm (4.4%). The volume change was 35.1 cm^3^ (13.7%). The corresponding CSAC sample shrank by 0.4 cm (2.5%) and 24.8 cm^3^ (9.7%), while the CAC sample shrank 0.3 cm (1.9%) and 17.2 cm^3^ (6.7%). No clear difference can be seen in the behavior of OPC, CAC and CSAC with 75% EP content. Samples containing 50% EP only shrank in the case of OPC and CSAC, while CAC samples did not change dimensions. Changing linear dimensions and volumes can be challenging when using these materials in practice. The hydration process and the associated temperature history significantly affect the autogenous shrinkage of concrete, which can lead to permanent dimensional changes after exposure to high temperatures^63^. This behavior is similar with other types of lightweight aggregates i.e. for CAC based aerogel composites^64^.

**Fig. 12:**
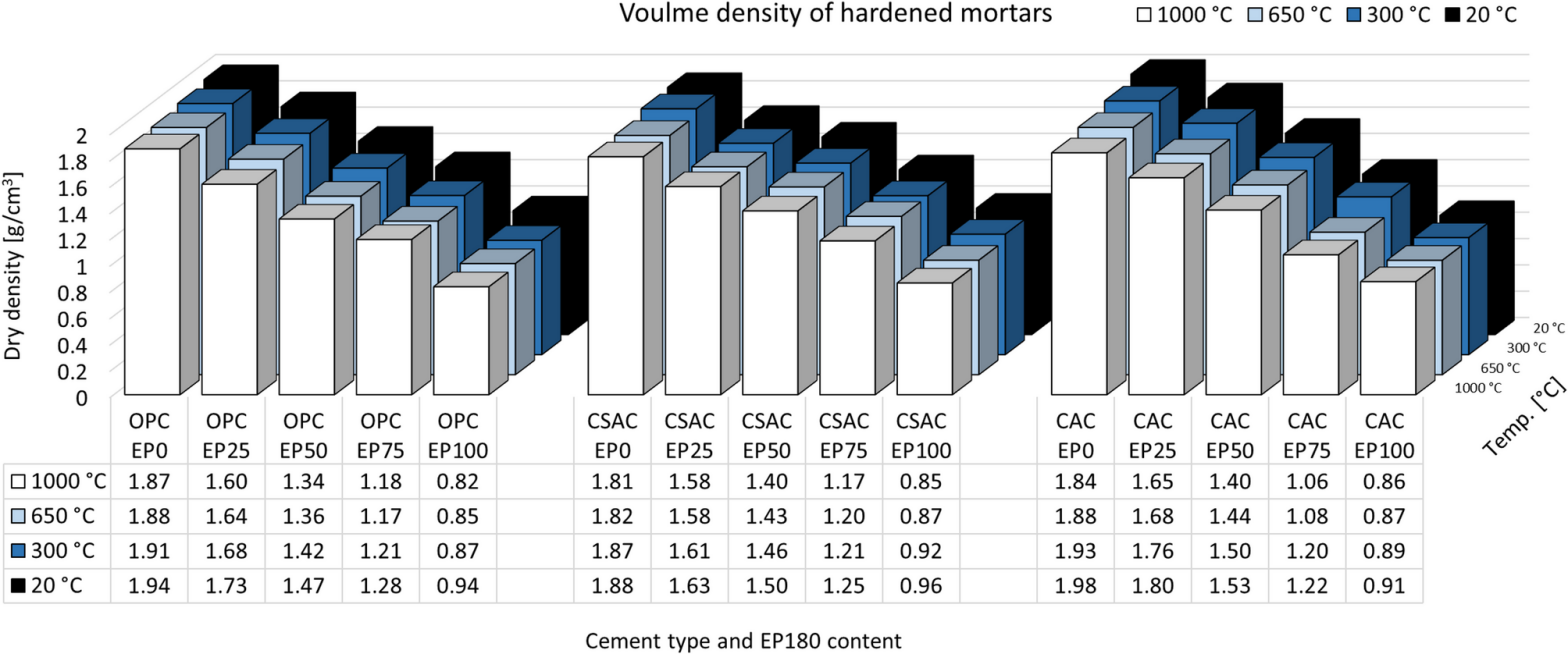
Volume density of hardened mortars.

The dry density of mortars that incorporate lightweight aggregates is significantly lower, with the reduction being directly proportional to the EP content. The results are illustrated in Fig. 12. For OPC-based mortars, the decrease in density with varying levels of sand replacement by EP (0%, 25%, 50%, 75%, and 100%) is measured at 1.94, 1.73, 1.47, 1.28, and 0.94 kg/m³, corresponding to reductions of 11%, 24%, 34%, and 52%, respectively, compared to the mortar without EP. This serves as an example, as other mortars formulated with different types of cement demonstrate similar trends. The general trend indicates that density decreases with increasing EP content and also with the temperature to which the mortars are exposed. This phenomenon is attributed to the loss of chemically bound water and the thermal decomposition of hydrates present in various cement pastes, as thoroughly discussed in the introduction, along with the rationale for the selected temperature ranges^28,40,41,47^. Although differences are observable among the various cements, they are not particularly significant. This is related to the shrinkage of samples subjected to heating, indicating that both mass loss and volume loss provided in Table 5 occur concurrently.

**Fig. 13:**
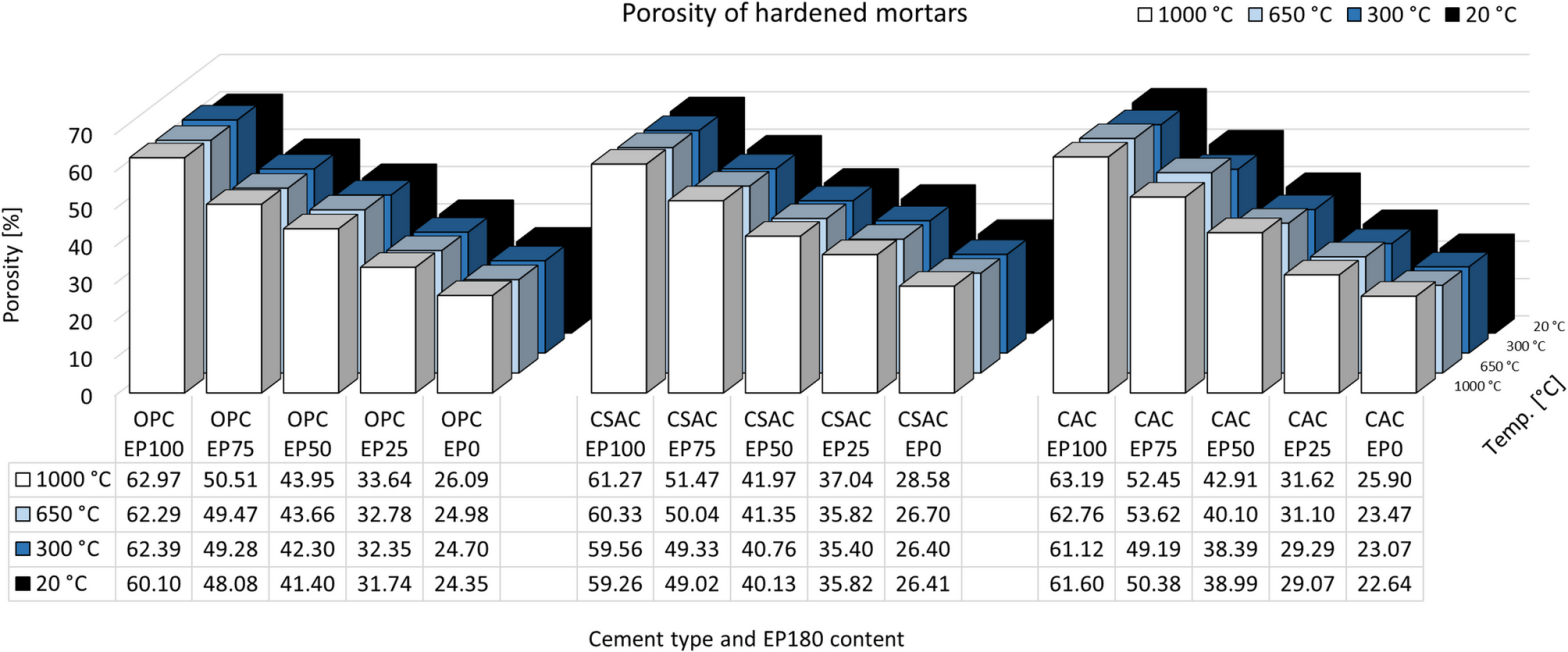
Total porosity of hardened mortars.

Similar relationships exist between samples when it comes to porosity, presented in Fig. 13. In general, porosity increases with heating temperature, although, as in the case of density, these changes are relatively small. It is connected to the general decrease of the dimensions of the samples. It was considered to include only the mass change without volume decrease, but it would be misleading. Instead of this the volumetric changes was included in the analysis. The true density is tested using the Le’Chateliers method, which takes into account only solid part of the material and is independent of original samples shape. From this reason the differences in porosity is also relatively small. Porosity was calculated using true and volume density ratio and not measured using MIP or other experimental method. Comparing different EP contents, of course, there is a major change in the porosity of mortars, which is due to the porosity of the aggregate itself. Porosity for all cements increases from about 22–28% without EP to about 59–63% with 100% EP. An interesting phenomenon, however, is the lowest porosity of CAC (22.64%) without perlite compared to OPC and CSAC (24.35% and 26.41%, respectively) and the highest porosity at 100% EP (CAC showed 61.60%, OPC 60.10%, and CSAC 59.26%). However, these are such minor differences that it is difficult to find their cause. In order to find it, perhaps an extensive analysis of pore size distribution would have to be prepared, but this is not the subject of this study and may be a future research objective.

**Fig. 14:**
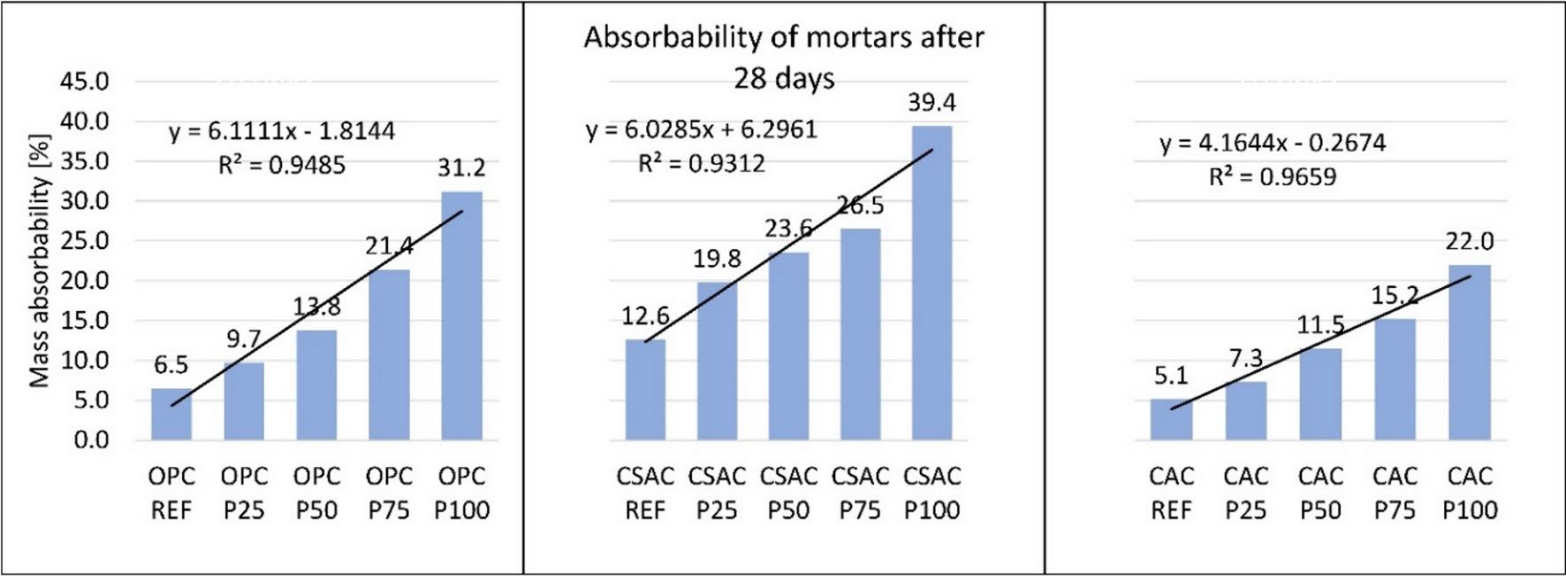
Absorbability of mortars.

Absorbability of mortars is closely related to their density and porosity. Absorbability is presented in Fig. 14. It is evident that an increase in perlite content leads to higher absorbability due to the increased porosity of the composites. While the densities and porosities of mortars made with different types of cement are comparable at 20 °C, the absorbability characteristics differ. For OPC based mortars, absorbability rises from 6.5% with no perlite to 31.2% when sand is fully replaced by EP. In contrast, CSAC exhibits a higher initial absorbability of 12.6%, reaching 39.4% for 100% EP (this is the highest recorded value in this research). The initial absorbability for CAC is 5.1%, increasing to 22% at 100% EP, which is the lowest absorbability recorded for this EP content across all samples. Since CAC exhibits the lowest absorbability, it is less susceptible to the ingress of aggressive chemical compounds, which may potentially enhance its durability^65^. Given that the densities of CAC and OPC mortars are nearly identical, the difference can be attributed to the open to closed porosity ratio, which is likely greater in OPC-based composites. Previous studies have reported lower permeability in CAC, which may further support these findings^66^. However, no additional research has been identified that specifically addresses lightweight mortars made from CAC or CSAC to corroborate these results. This is an area open to future testing, since it is connected to pore size distribution as well. Figure 14 further depicts the excellent correlation between absorbability and EP content for all three types of cement, revealing linear relationships with R² values ranging from 0.93 to 0.97.

### Thermal properties of mortars

**Fig. 15:**
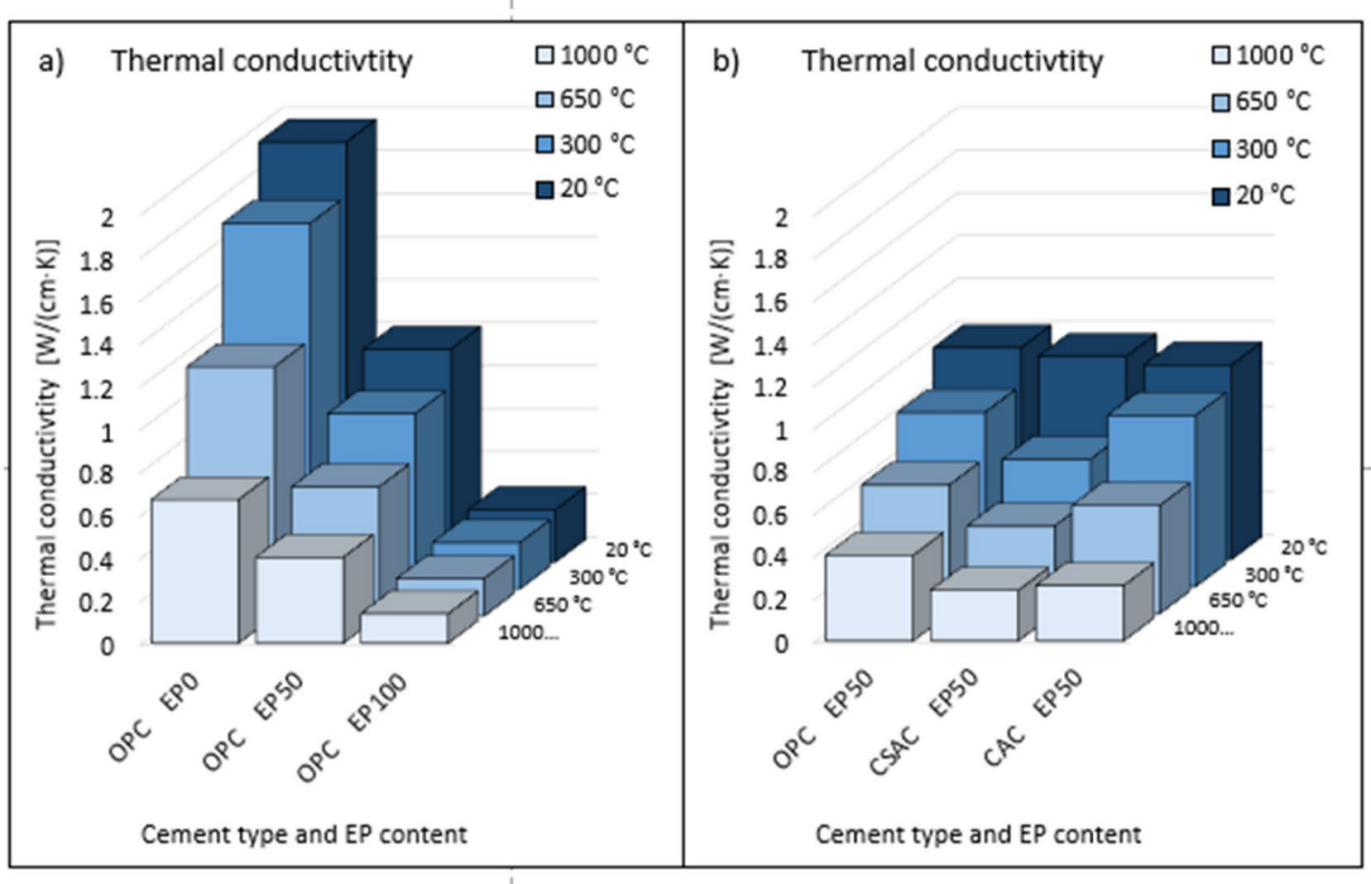
Thermal conductivity coefficient of mortars, (**a**) for OPC with various EP content, (**b**) for OPC, CSAC, CAC, with constant 50% EP content.

**Fig. 16:**
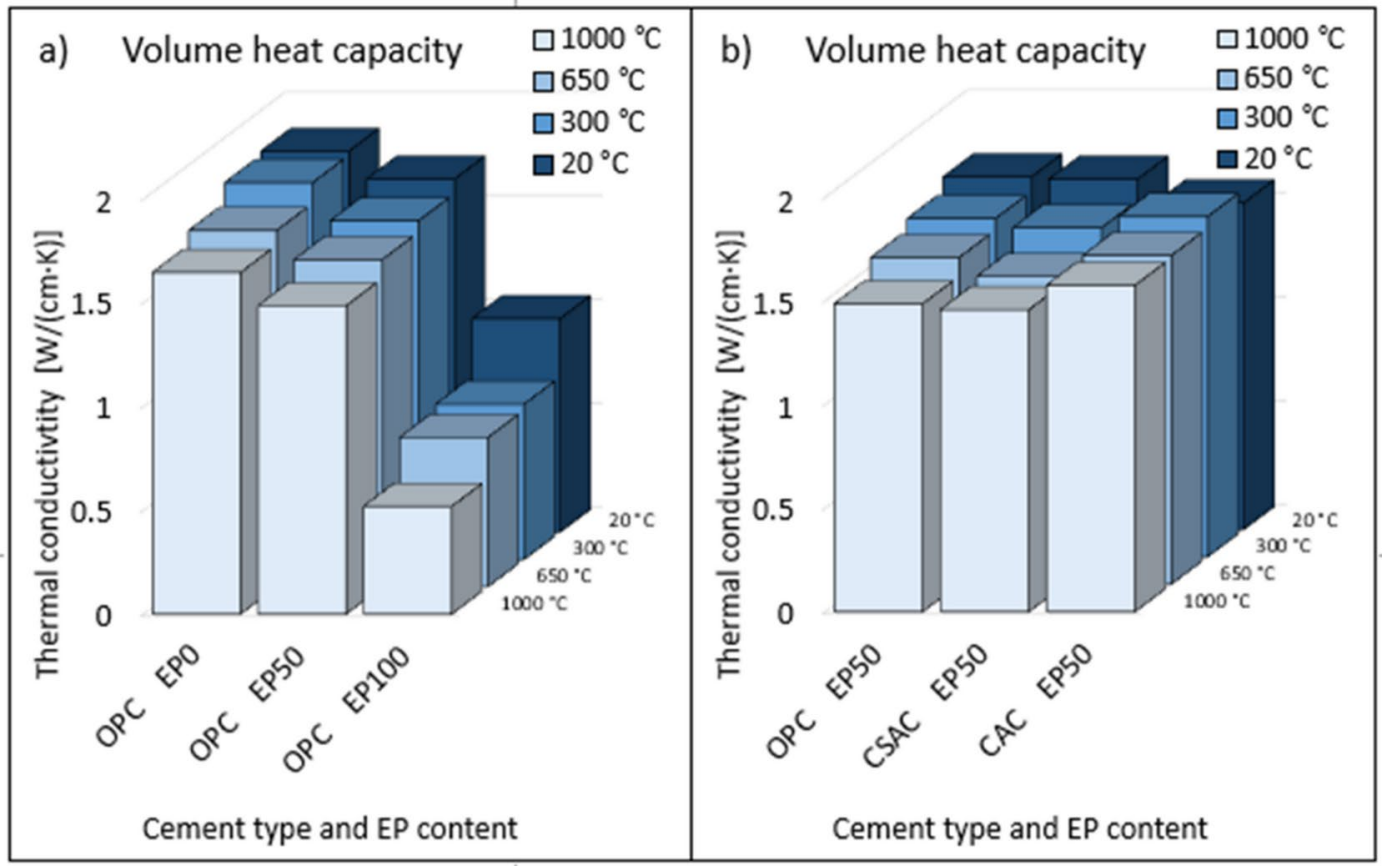
Volume heat capacity of mortars, (**a**) for OPC with various EP content, (**b**) for OPC, CSAC, CAC, with constant 50% EP content.

Thermal properties, namely thermal conductivity (Fig. 15) and heat capacity (Fig. 16) of mortars were examined for selected examples.

First, mortars with OPC containing 0%, 50% and 100% EP were examined, without heating and after heating to 300 °C, 650 °C and 1000 °C (Fig. 15a)). Thermal conductivity obviously decreased with increasing EP content. In this case, the decrease was proportional. Thermal conductivity also decreased after heating and was the lower the higher the heating temperature was. The effect was also proportional, but was most pronounced in the absence of EP in the mortar formulation. In the case of EP 100%, the decrease occurred, but was small due to the small absolute values. In the case of heat capacity (Fig. 16a)), a decrease with temperature was also evident, albeit relatively small. A noticeable difference was observed in the decrease with EP content. This was not a linear decrease, and a greater difference was seen between 50% and 100% EP than between 0% and 50%, in which case the decrease was small. The second comparison was between mortars with different cements with the same EP content (Fig. 15b)). For CAC and CSAC, as the heat treatment temperature increased, the thermal conductivity decreased as in the case of OPC. Thermal conductivity after heating was ruled by similar relationships as compressive strength. For CAC and OPC it decreased uniformly, and for CSAC the most pronounced change was between 20 °C and 300 °C. At normal temperature (20 °C) the differences were small and arranged in the sequence OPC-CSAC-CAC, at other temperatures the arrangement was as follows: OPC-CAC-CSAC, with the conductivity of OPC similar to CAC and much higher than CSAC. A comparison between cements with 50% EP content (Fig. 16b)) showed almost identical results regardless of temperature. The observed differences in heat capacity as a function of heating temperature were small. While the heat capacity of concrete can remain relatively stable, the thermal conductivity may not exhibit the same consistency, especially when subjected to varying temperatures^67^. Lightweight aggregates can significantly reduce the thermal conductivity of concrete while maintaining a comparable heat capacity^68^.

**Fig. 17:**
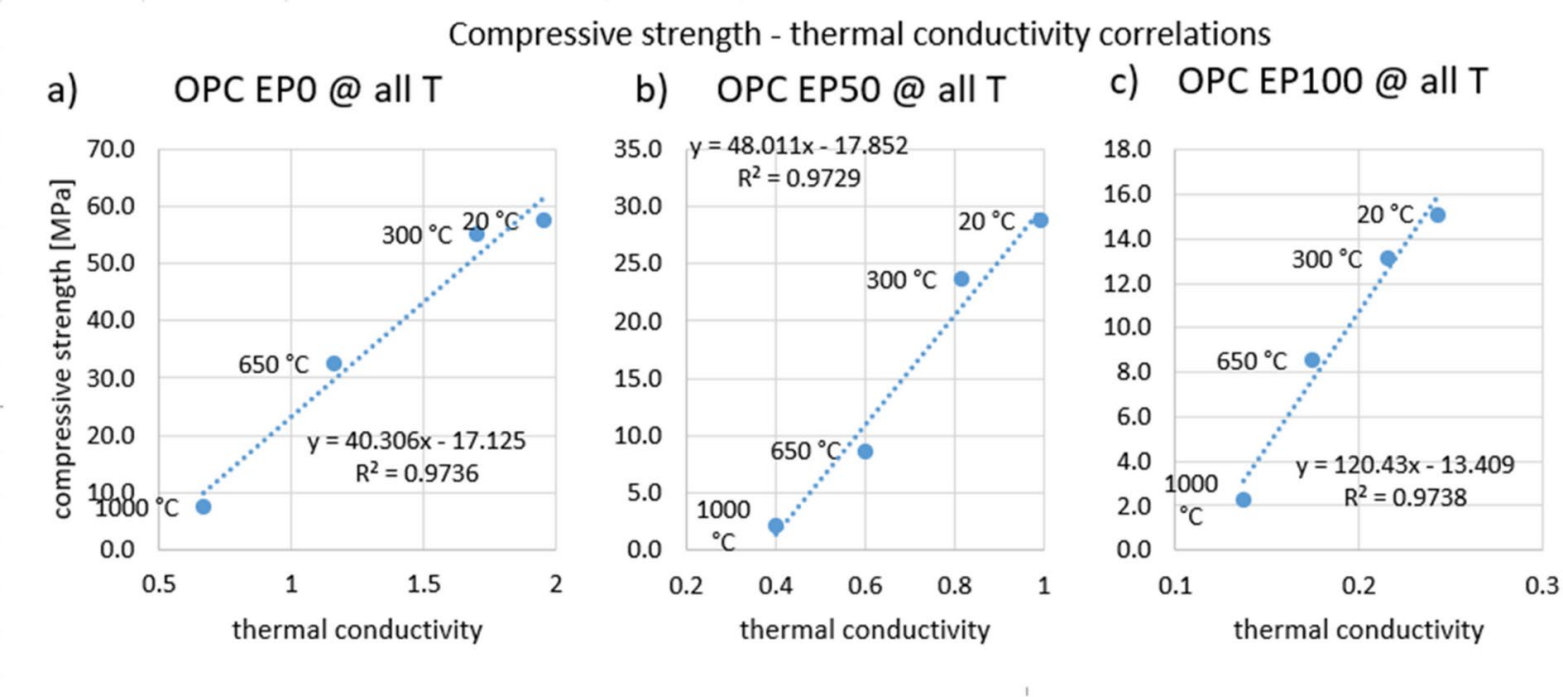
Correlation between compressive strength and thermal conductivity for OPC after heating to all temperatures for expanded perlite content (**a**) 0%, (**b**) 50%, (**c**) 100%.

**Fig. 18:**
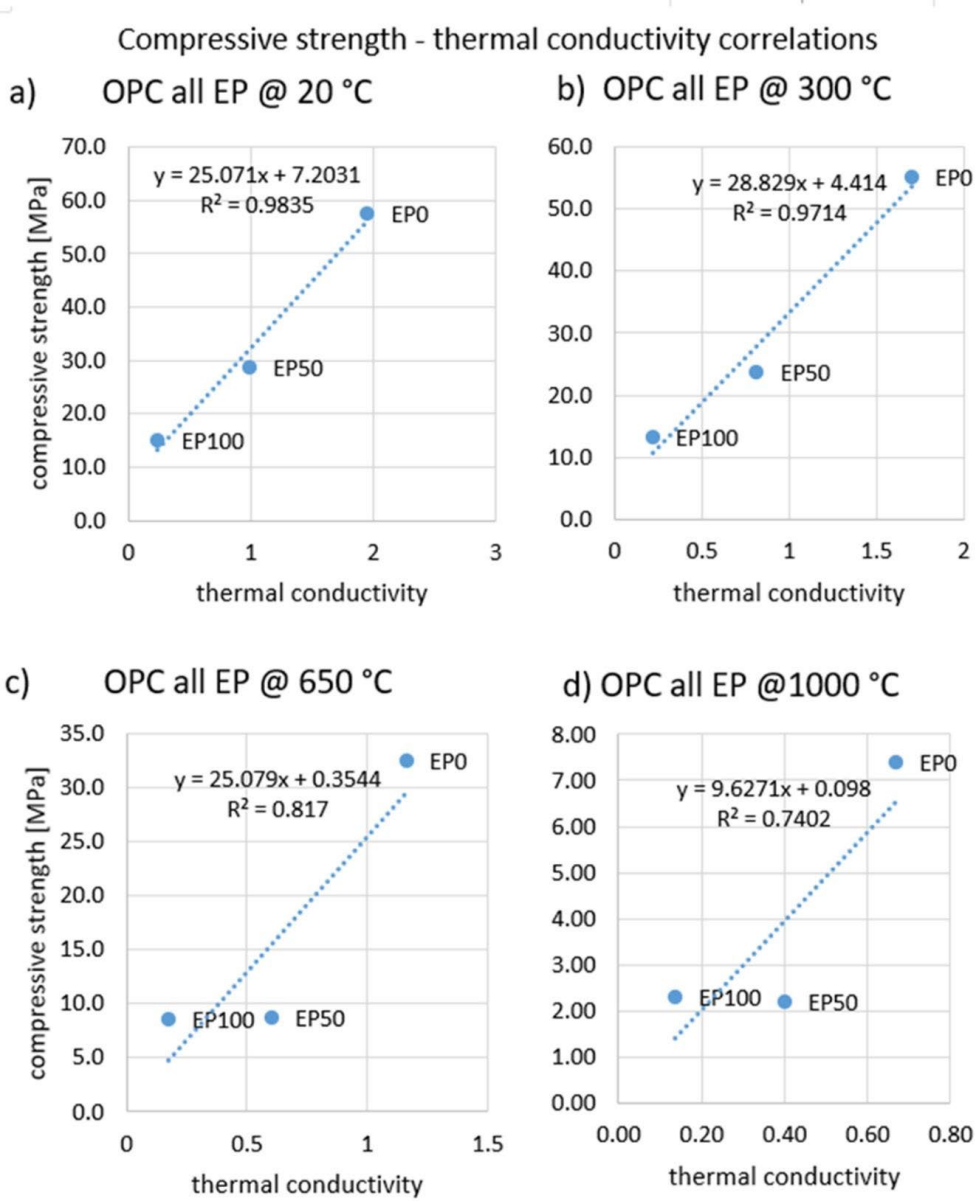
Correlation between compressive strength and thermal conductivity for OPC at EP replacement levels after heating up to temperatures (**a**) 20 °C, (**b**) 300 °C, (**c**) 650 °C, (**d**) 1000 °C.

**Fig. 19:**
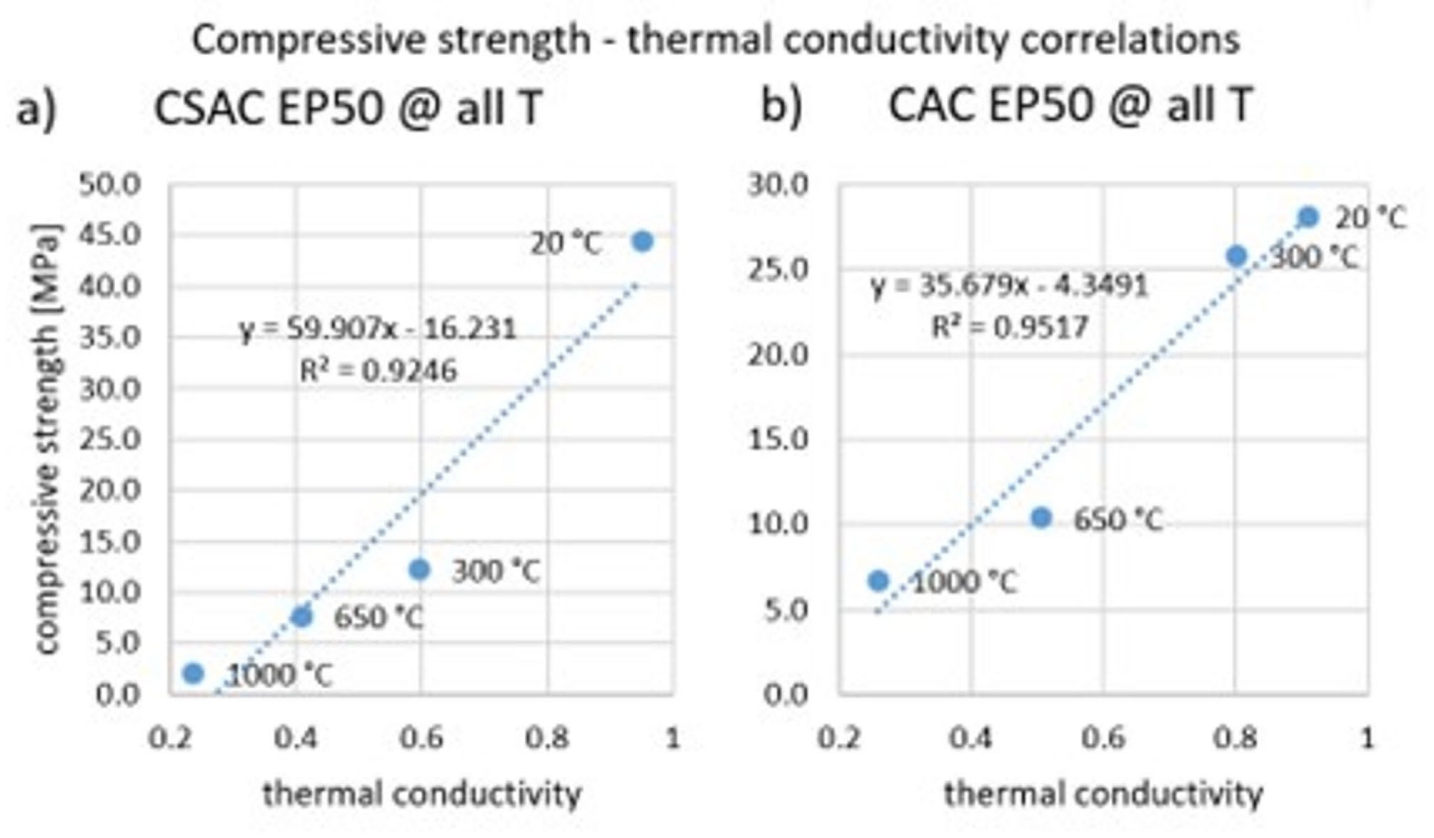
Correlation between compressive strength and thermal conductivity for (**a**) CSAC after heating to all temperatures for 50% expanded perlite content, (**b**) CAC after heating to all temperatures for 50% expanded perlite content.

Graphs of three groups of correlations were prepared. The first shows correlation between compressive strength and thermal conductivity separately for OPC containing 0%, 50% and 100% EP (Fig. 17). Temperature was the variable. The R^2^ value is around 0.97 for each case, indicating a very good correlation. The second comparison of compressive strength with thermal conductivity was made for OPC separately for different temperatures (Fig. 18). The EP content was the variable. For temperatures of 20 °C and 300 °C, an excellent correlation was also shown, as evidenced by an R^2^ value above 0.97. After heating to 650 °C, the R^2^ coefficient was 0.82, and after heating to 1000 °C it was 0.74, indicating a worse correlation. In the latter two cases, the graph suggests that the measurement should be supplemented with at least two more results to get a more reliable correlation. The third comparison of compressive strength with thermal conductivity was performed for CAC and CSAC containing 50% EP (Fig. 19). Again, temperature was the variable. The R^2^ values were above 0.92, indicating a very good correlation.

### Macroscopic observations of mortars

**Fig. 20:**
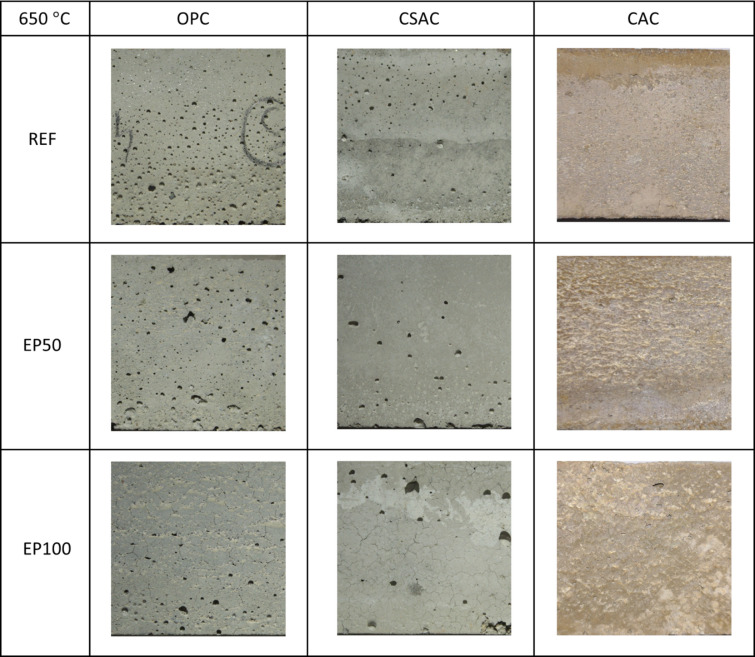
Macro photographs of surfaces of mortars subjected to 650 °C. Photographed area 40 × 40 mm.

**Fig. 21:**
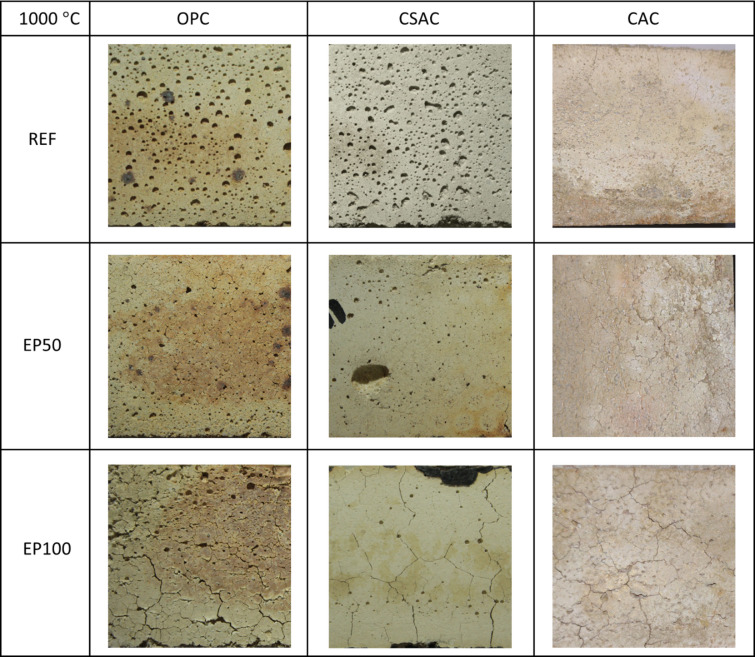
Macro photographs of surfaces of mortars subjected to 1000 °C. Photographed area 40 × 40 mm.

Sample surfaces were photographed to illustrate thermal cracks after heating to 650 °C (Fig. 20) and 1000 °C (Fig. 21), and subsequent cooling. No visible changes were apparent to the naked eye after heating to 300 °C. Cracks became noticeable after heating to 650 °C; however, they were very fine and difficult to detect. Only a few cracks appeared on the surfaces of reference mortars without EP, primarily located at the edges. In contrast, mortars containing EP exhibited more cracks on their surfaces. These cracks were longer and, while slightly thicker than those on the reference mortars, they remained quite thin.

**Fig. 22:**
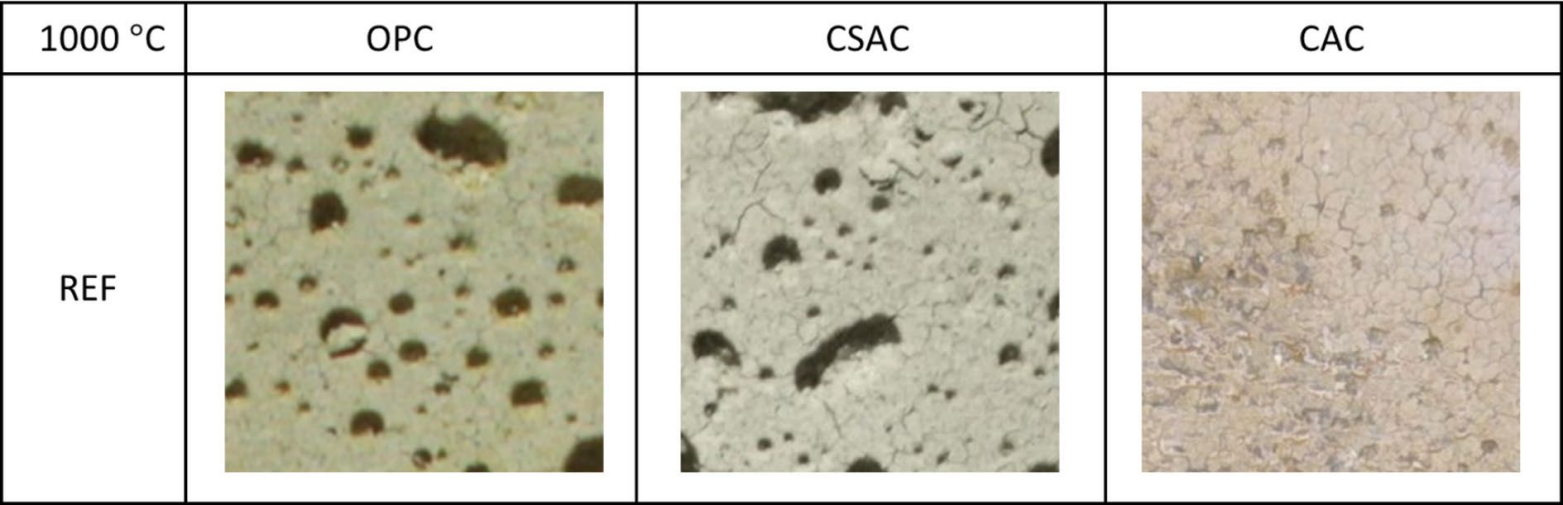
Close-ups of surfaces of REF mortars subjected to 1000 °C. Photographed area 40 × 40 mm. Similar pattern of very fine cracks was visible on EP25, EP50 and EP75 subjected to 1000 °C. EP75 additionally exhibited thicker cracks similar to EP100.

When heated to 1000 °C, noticeable differences in color were observed between OPC and CSAC. The OPC appeared more yellowish or beige, while the CSAC acquired a lighter hue. No color change was detected in CAC mortars. Cracks were present on the surfaces of all samples, but in the case of the reference samples, the cracks were barely noticeable, requiring bigger magnification (Fig. 22). Similar findings have been reported in studies involving composites, specifically alkali-activated slag mortars^69^ and normal-weight cement mortars containing waste steel slag and waste clay brick^70^.

### Microscopic observations of mortars

Samples of mortars were observed through a Scanning Electron Microscope. Samples chosen for observations were those of all cements with 50% of EP content cured and tested in ambient temperature and those heated to 650 °C.

**Fig. 23:**
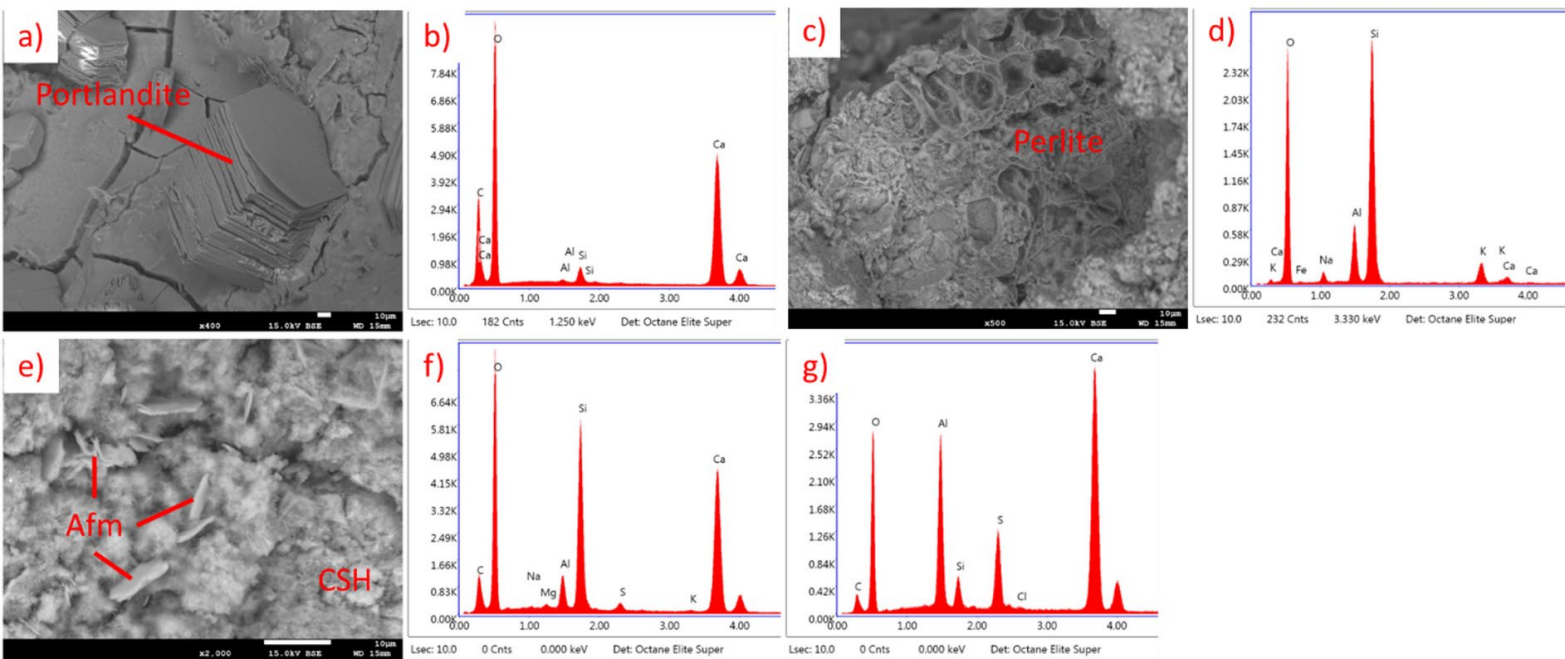
SEM images and EDS spectra for OPC EP 50, cured at 20 °C, not subjected to heating. (**a**) SEM image of portlandite, (**b**) EDS spectrum of portlandite, (**c**) SEM image of perlite grain, (**d**) EDS spectrum of perlite grain, (**e**) SEM image of Afm and CSH phases, (**f**) EDS spectrum of CSH phase, (**g**) EDS spectrum of AFm phase.

**Fig. 24:**
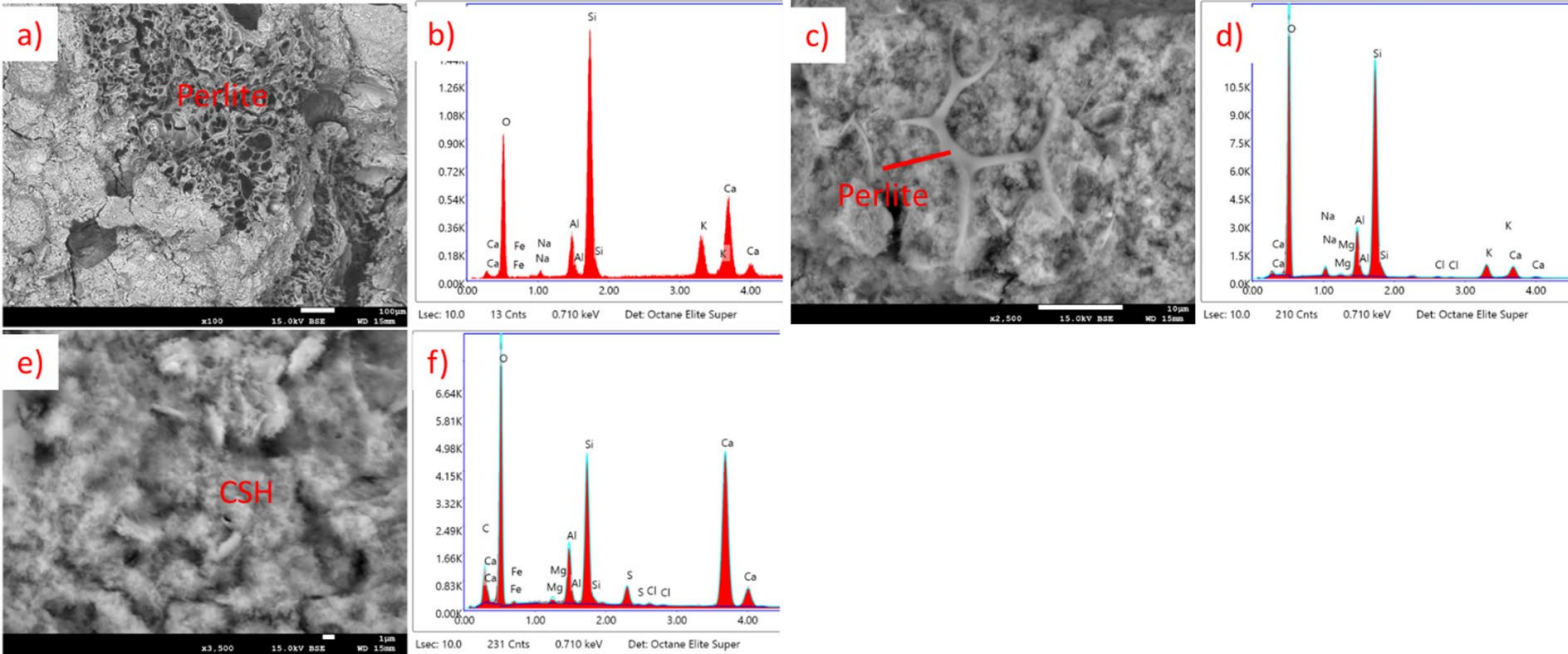
SEM images and EDS spectra for OPC EP 50, subjected to heating at 650 °C. (**a**) SEM image of perlite grain, (**b**) EDS spectrum of perlite grain, (**c**) SEM image of perlite fragment, (**d**) EDS spectrum of perlite fragment, (**e**) SEM image of CSH phase, (**f**) EDS spectrum of CSH phase.

For OPC results are presented in Fig. 23 (20 °C) and Fig. 24 (650 °C). In the sample not subjected to heating the CSH phase, portlandite and Afm phase are visible. This sample is representative for lightweight mortar containing EP. In the sample after heating, CSH phase remains, but portlandite and Afm phase are not noticeable. This is connected to thermal decomposition of both of those compounds below 650 °C^40–43^. Figure 24c) presents a structure that seem to be melted perlite fragment or silica fume, but since the temperature is way lower than the melting point of those materials it is probably only a detached fragment of perlite, with such appearance.

**Fig. 25:**
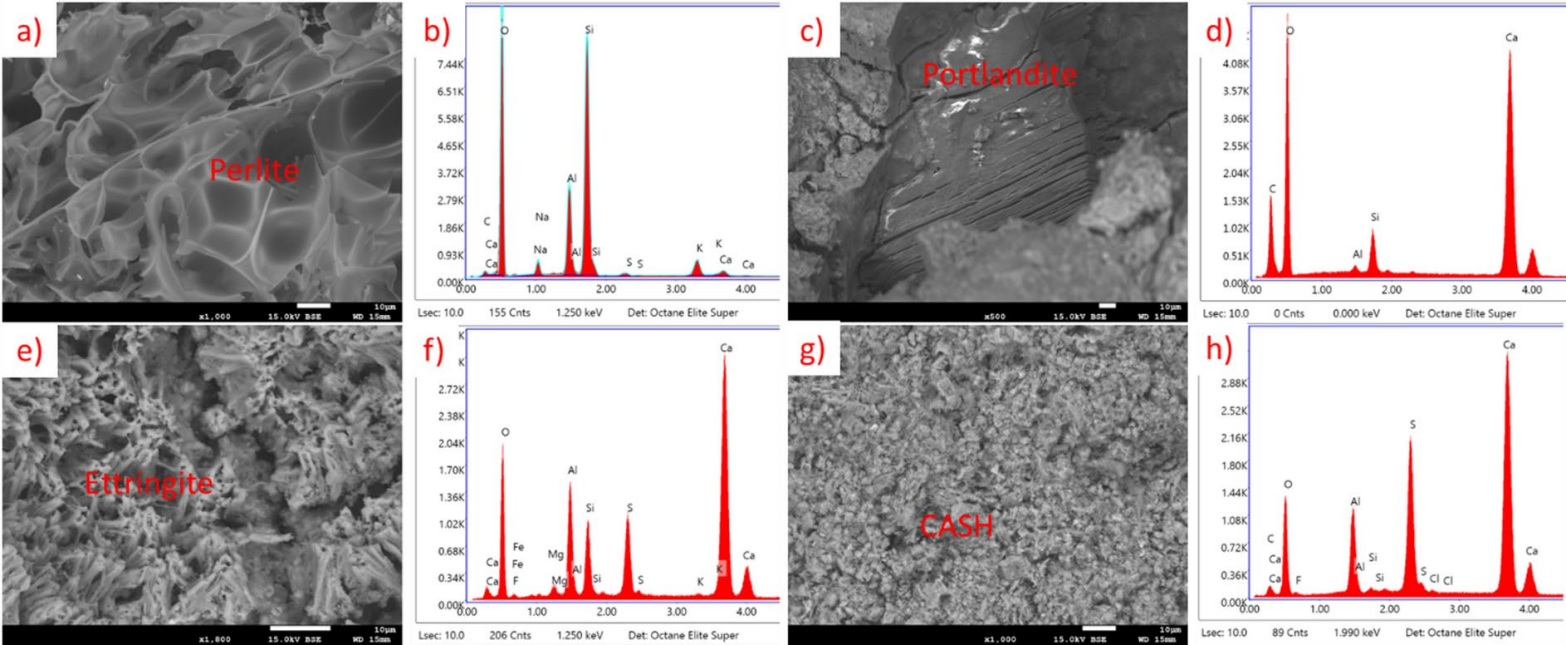
SEM images and EDS spectra for CSAC EP 50, cured at 20 °C, not subjected to heating. (**a**) SEM image of perlite grain, (**b**) EDS spectrum of perlite grain, (**c**) SEM image of portlandite, (**d**) EDS spectrum of portlandite, (**e**) SEM image of ettringite, (**f**) EDS spectrum of ettringite, (**g**) SEM image of CASH phase, (**h**) EDS spectrum of CASH phase.

**Fig. 26:**
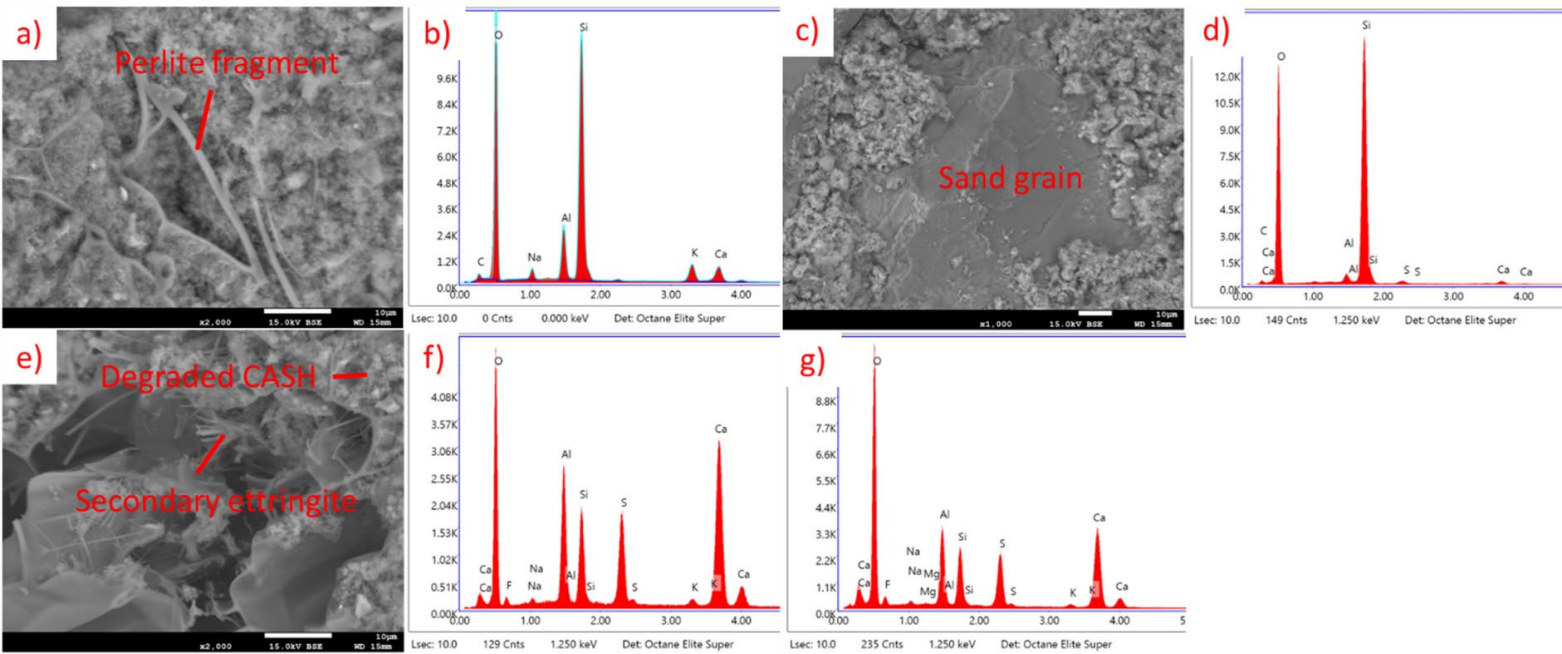
SEM images and EDS spectra for CSAC EP 50, subjected to heating at 650 °C. (**a**) SEM image of perlite fragment, (**b**) EDS spectrum of perlite, (**c**) SEM image of sand grain, (**d**) EDS spectrum of sand grain, (**e**) SEM image of degraded or secondary CASH phase and secondary ettringite, (**f**) EDS spectrum of degraded or secondary CASH phase, (**g**) SEM image of secondary ettringite.

For CSAC, results are presented in Fig. 25 (20 °C) and Fig. 26 (650 °C). In the sample not subjected to heating the CASH phase, ettringite and portlandite are visible. In the sample after heating, CASH phase remains, but in degraded form. The portlandite and the ettringite are not present, although it seems that some moisture ingress occurred in the meantime before SEM observations and some secondary ettringite was produced. This absence of portlandite and ettringite is connected to thermal decomposition of both of those compounds below 650 °C^40–42,44^.

**Fig. 27:**
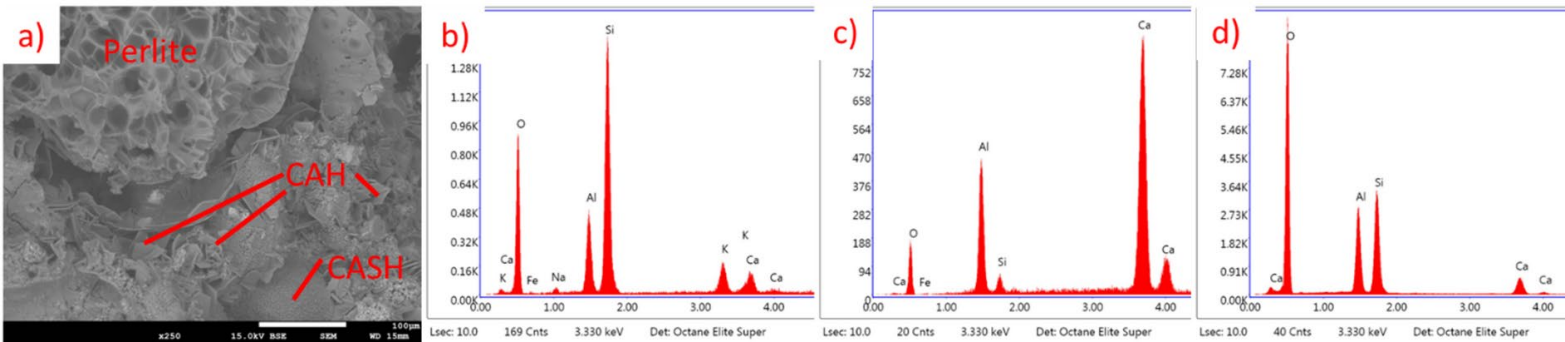
SEM images and EDS spectra for CAC EP 50, cured at 20 °C, not subjected to heating. (**a**) SEM image of perlite grain surrounded by hydration products, (**b**) EDS spectrum of perlite, (**c**) EDS spectrum of CAH phase, (**d**) EDS spectrum of CASH phase.

**Fig. 28:**
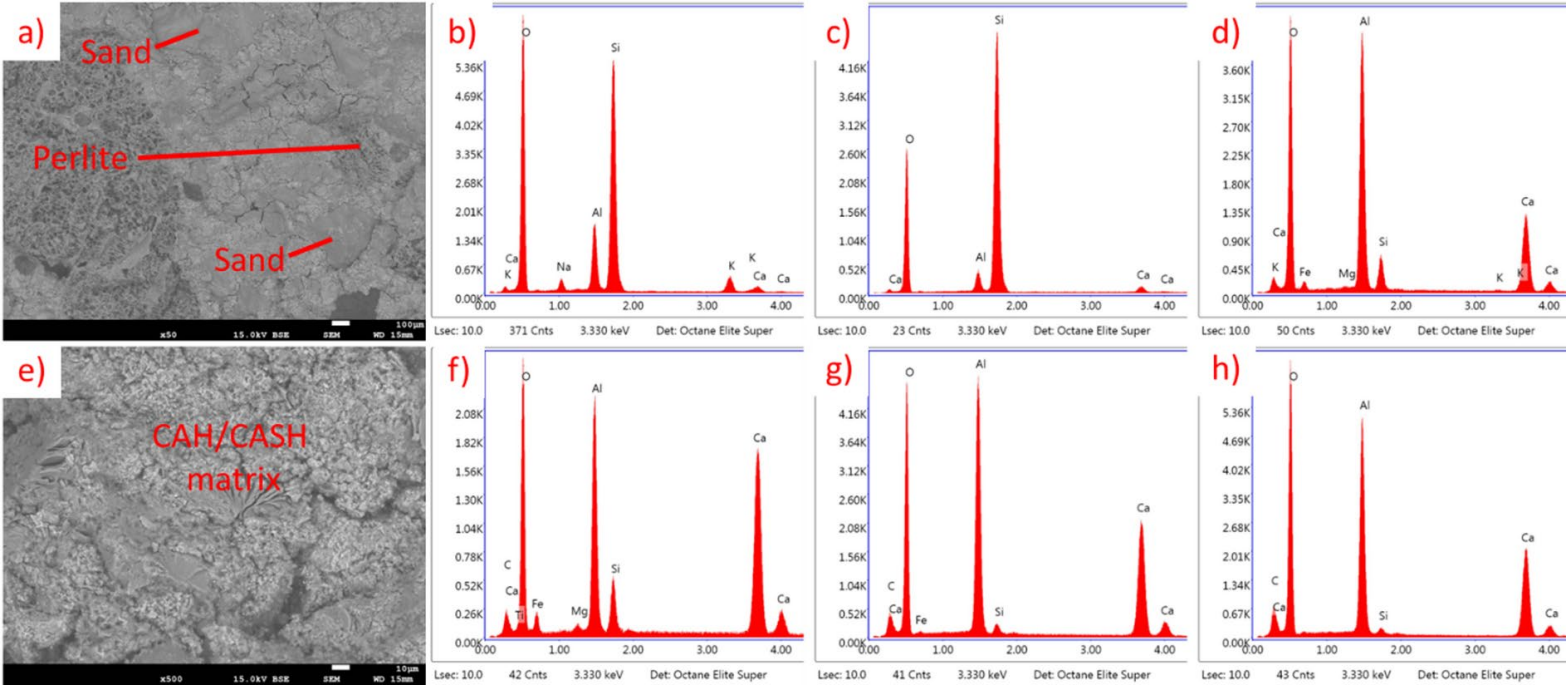
SEM images and EDS spectra for CAC EP 50, subjected to heating at 650 °C. (**a**) SEM image of perlite and sand grains surrounded by hydration products, (**b**) EDS spectrum of perlite, (**c**) EDS spectrum sand, (**d**) EDS spectrum of CAH/CASH phase, (**e**) SEM image of CAH/CASH phase, (**f**–**h**) EDS spectrum of various areas of CAH/CASH in different proportions.

For CAC, results are presented in Fig. 27 (20 °C) and Fig. 28 (650 °C). At temperatures around 20 °C, the primary hydration products of CAC include metastable low-density phases such as CAH_10_ and C_2_AH_8_. At elevated temperatures, the behavior of these phases changes significantly. The transformation from the low-density phases to the high-density phase (C_3_AH_6_) is accelerated, leading to an increase in porosity and a corresponding decrease in compressive strength^71–73^. It is also connected with a higher Ca/Al ratio, which is visible in Figs. 27 and 28.

## Conclusions

The primary factor influencing the consistency of the mix is the type of cement used when the perlite content is low, whereas the perlite itself becomes the dominant factor when its content is higher.

In terms of compressive strength, the OPC- and CAC-based mortars exhibited comparable performance up to 650 °C. The CSAC-based ones showed worse properties. The highest compressive strength after heating to the temperature of 1000 °C was found for CAC. Other mortars showed similar behavior—their residual strength was comparable.

The behavior of mortars depending on the type of cement, EP content and heating temperature can be easily and reliably modeled. For density, absorbability and consistency, there are simple linear relationships. In the case of compressive strength, however, care must be taken in selecting the type of correlation. Exponential correlation between compressive strength and EP content across all types of cement gives better results compared to linear correlation. The analysis shows that for OPC and CAC, polynomial correlation can be taken as the best representation of the strength-temperature relationship. The exponential and logarithmic models yield worse results. The situation is different for CSAC. The logarithmic model gave an R^2^ value above 0.99 for each EP content. Polynomial and exponential models were also efficient.

There are large differences in thermal conductivity depending on the type of cement, EP content and heating temperature. The relationships are similar to those for compressive strength. Thermal capacity is independent of the type of cement used and the heating temperature but is closely related to EP content. The performed correlation analysis showed that by controlling compressive strength change related to the EP content, the thermal properties of mortars with different EP contents subjected to heating to different temperatures can be predicted. A correlation model of strength and conductivity for temperatures of 650 °C and 1000 °C needs to be developed.

Considering the behavior of individual mortars and the interrelationships between the various cements and EP contents, mortars containing finer EP (from previous studies^5^ show similar behavior to those used for the current study. The differences are quantitative, and mortars containing finer EP show higher strengths in groups of individual cements as well as comparing the same amounts of finer and coarser EP. The relations between density, absorbability and consistency are similar as well.

## Data Availability

The datasets generated and analysed during the current study are available from the first author Jan Pizoń on reasonable request.
